# Distinct Functions of *Bombyx mori* Peptidoglycan Recognition Protein 2 in Immune Responses to Bacteria and Viruses

**DOI:** 10.3389/fimmu.2019.00776

**Published:** 2019-04-12

**Authors:** Liang Jiang, Weiqiang Liu, Huizhen Guo, Yinghui Dang, Tingcai Cheng, Wanying Yang, Qiang Sun, Bingbing Wang, Yumei Wang, Enyu Xie, Qingyou Xia

**Affiliations:** ^1^Biological Science Research Center, Southwest University, Chongqing, China; ^2^Chongqing Key Laboratory of Sericultural Science, Chongqing Engineering and Technology Research Center for Novel Silk Materials, Southwest University, Chongqing, China; ^3^Guangdong Provincial Key Laboratory of Agro-Animal Genomics and Molecular Breeding, South China Agricultural University, Guangzhou, China

**Keywords:** DAP-type PGN, PGRP, Imd, silkworm, BmNPV, PTEN, Akt, apoptosis

## Abstract

Peptidoglycan recognition protein (PGRP) is an important pattern recognition receptor in innate immunity that is vital for bacterial recognition and defense in insects. Few studies report the role of PGRP in viral infection. Here we cloned two forms of PGRP from the model lepidopteran *Bombyx mori*: BmPGRP2-1 is a transmembrane protein, whereas BmPGRP2-2 is an intracellular protein. BmPGRP2-1 bound to diaminopimelic acid (DAP)-type peptidoglycan (PGN) to activate the canonical immune deficiency (Imd) pathway. *BmPGRP2-2* knockdown reduced *B. mori* nucleopolyhedrovirus (BmNPV) multiplication and mortality in cell lines and in silkworm larvae, while its overexpression increased viral replication. Transcriptome and quantitative PCR (qPCR) results confirmed that *BmPGRP2* negatively regulated *phosphatase and tensin homolog* (*PTEN*). *BmPGRP2-2* expression was induced by BmNPV, and the protein suppressed PTEN-phosphoinositide 3-kinase (PI3K)/Akt signaling to inhibit cell apoptosis, suggesting that BmNPV modulates BmPGRP2-2-PTEN-PI3K/Akt signaling to evade host antiviral defense. These results demonstrate that the two forms of BmPGRP2 have different functions in host responses to bacteria and viruses.

## Introduction

Innate immunity is a self-defense mechanism against infectious non-self entities and is present in all metazoans (1, 2). The innate immune system of insects consists of humoral defenses that include the production of soluble effector molecules and cellular response like phagocytosis and encapsulation that are mediated by hemocytes (3). The innate immune response is mediated by germline-encoded pattern-recognition receptors (PRRs) that recognize pathogen-associated molecular patterns (PAMPs) that are present in pathogens but absent in the host (2, 4, 5). PAMPs include b-glucan, lipopolysaccharides (LPS) of gram-negative (G–) bacteria, and peptidoglycans (PGNs) of both gram-positive (G+) and G– bacteria, as well as bacterial and viral DNA and RNA and related molecules (2, 4, 5). LPS activates the expression of antimicrobial peptides (AMPs) in *Manduca sexta* (6) and malpighian tubules *of Drosophila* (7). PGN—an essential cell wall component in most bacteria—stimulates various immune reactions in insects (2, 5, 8). Lysine (Lys)-type PGNs are found in many G+ bacteria, whereas diaminopimelic acid (DAP)-type PGNs are found in G– and some G+ bacteria (5, 9, 10). Multiple PRRs have been identified in invertebrates, such as lectin, hemolin, G– binding proteins (GNBPs), Toll-like receptors (TLRs), and PGN recognition proteins (PGRPs); the latter two have been shown to recognize PGN (2, 5, 10).

PGRP was first purified from the hemolymph of silkworm *Bombyx mori* (11). Since then, nearly 100 PGRP family members have been identified from insects to mammals. These proteins are highly conserved and have a PGRP domain that is similar to the bacteriophage T7 lysozyme, an *N*-acetylmuramyl-alanine amidase (9, 12, 13). Insect PGRPs are categorized as short (PGRP-S), which are small extracellular proteins with signal peptides, or long (PGRP-L), which have long transcripts and are intracellular, extracellular, or transmembrane proteins (12, 14). Some PGRP-Ls have multiple splice forms; for example, the 13 *Drosophila* PGRP genes encode 19 proteins and the seven genes in mosquito (*Anopheles gambiae*) encode nine proteins (5, 12, 15, 16).

PGRPs have multiple immune-related functions. In *Drosophila*, PGRP-SB1 and -SB2, -SC1A/1B/2, and -LB have zinc-dependent amidase activity involving the removal of peptides from glycan chains to inhibit or scavenge the biological activity of PGN. Some of these PGRPs modulate the host immune response by eliminating PGN (8, 17–20). PGRP-SA, -SD, -LA, -LC, -LD, -LE, and -LF lack zinc-binding residues required for amidase activity while retaining the capacity to bind and recognize PGN (8, 18). Other PGRPs lacking amidase activity mediate PGN-dependent activation of the prophenoloxidase (proPO) cascade (21, 22) in which ProPO is activated to PO, leading to melanization that is toxic to microorganisms (11, 23). Transmembrane PGRP-LC and -LE recognize DAP-type PGNs and activate the immune deficiency (Imd) pathway. The former binds PGN and interacts with Imd protein via the extracellular PGRP domain and an intracellular domain, respectively (15, 24, 25). PGRP-LB has amidase activity to cleave DAP-type PGNs, limiting availability of ligand for PGRP-LC and thus inhibiting the Imd pathway (26). PGRP-SA is an extracellular protein that recognizes Lys-type PGNs and activates Toll signaling. The serine protease cascade is triggered when PGRP-SA binds to PGN, which cleaves the inactive pro Spatzle (Spz) protein to an active form, which in turn binds to and activates Toll receptor (8, 27–31).

Imd and Toll signaling activation induces AMPs against invading bacteria and fungi (5, 8, 14, 27, 32). The GATA factor Serpent is required for the onset of humoral defenses in *Drosophila* embryos (33). In the silkworm, the Imd pathway can be activated by *Escherichia coli* (*E. coli*) and *Serratia marcescens* (*S. marcescens*) (34), whereas the Toll pathway could be induced by *Bacillus bombysepticus* (35), *Nosema bombycis* (36), and *Beauveria bassiana* (34). Some studies have shown that these pathways are also involved in the antiviral immune response (37–41). In *Drosophila*, the replication of the RNA viruses cricket paralysis virus (CrPV) and alphavirus is increased by mutation of Imd pathway components (37, 38). Activation of Toll signaling inhibits dengue virus in *Aedes aegypti* (40); Toll-7 interacts with vesicular stomatitis virus and induces antiviral autophagy independently of canonical Toll signaling (39).

The model lepidopteran *B. mori* is an important insect because of its production of silk (42–44); as such, infection by pathogenic bacteria, fungi, and viruses can cause serious economic losses. *B. mori* nucleopolyhedrovirus (BmNPV) is the major threat to silkworms (45, 46). A genome analysis revealed 12 PGRP genes in *B. mori* (47), some of which the function has been studied. Five BmPGRP-S showed strong amidase activity toward DAP-PGN (48). BmPGRP-S4 (49) and BmPGRP-S5 (50) bound PGNs to increase proPO activation. *BmPGRP-S3* could be induced by *B. mori* cytoplasmic polyhedrosis virus (BmCPV) (51). Recently, our study showed that *BmPGRP-S2* was up-regulated upon BmCPV infection (52), overexpression of which can activate the Imd pathway and induce increased AMPs to enhance the antiviral capacity of transgenic silkworm against BmCPV (53). In this study, we cloned two forms of BmPGRP2: BmPGRP2-1 bound to DAP-type PGN to activate Imd signaling, whereas BmPGRP2-2 was induced by BmNPV and negatively regulated the phosphatase and tensin homolog (PTEN)-phosphoinositide 3-kinase (PI3K)/Akt pathway to inhibit cell apoptosis. Our results demonstrate that the two forms of BmPGRP2 have distinct functions in the host response to pathogenic bacteria and viruses. The present study confirms that PGRP was induced by viruses to escape host antiviral immunity.

## Materials and Methods

### Silkworm Strain, Cell Lines, and Viruses

*B. mori* strains Dazao (DZ) and 932 were maintained at the Gene Resource Library of Domesticated Silkworm (Southwest University, Chongqing, China). BmE and BmN4-SID1 cell lines (54) were cultured at 27°C. BmNPV (Guangdong strain) and BmNPV expressing green fluorescent protein (BmNPV-GFP) were collected from the haemolymph of infected silkworm larvae and the infected BmE cells, respectively (55, 56).

### Cloning, Reverse Transcription (RT-)PCR, and Quantitative (q)PCR Analysis

Based on bioinformatics analysis, primers were designed to amplify the open reading frame (ORF) of *BmPGRP2* (BmPGRP2-1 ORF and BmPGRP2-2 ORF) by PCR. The 3′ untranslated region (UTR) was amplified by 3′ rapid amplification of cDNA ends. Six forward primers (5F-1,-2,-3,-4,-5, and-6) and two reverse primers (5R-1 and-2) were used to amplify the 5′ UTR. The eggs of 2-, 4-, 6-, and 8-day-old, hatched silkworm, first instar molt, second instar, second instar molt, third instar, third instar molt, fourth instar, fourth instar molt, fifth instar larvae, pupae of 2-, 4-, 6-, and 8-day-old, and moth of DZ silkworms were used for RNA extraction using Total RNA Kit (#R6834-01 and R6934-01, Omega, USA). The RNA of head, cuticle, hemocyte, midgut, fat body, silk gland, trachea, malpighian, ovary, and testis of female and male silkworms (DZ) was extracted at day-3 fifth instar. The total RNA was treated with RNase-Free DNase I (#M6101, Promega, USA) and then reverse transcribed into cDNA using M-MLV Reverse Transcriptase (#M1701, Promega, USA). The cDNA temples of different developmental stages were used for RT-PCR with primers BmPGRP2-1qRT, BmPGRP2-2qRT, and internal control *TIF-4A* (53, 57–59), of which the amplification cycles was 30, 30, and 25, respectively. The cDNA of each tissue was used for qPCR analysis with primers BmPGRP2-1qRT and BmPGRP2-2qRT on an ABI Prism 7500 (Applied Biosystems, USA) using an SYBR Premix Ex Taq II (#RR820A, TaKaRa, China). The thermal program of qPCR consisted of 95°C for 30 s, 40 cycles at 95°C for 5 s and 60°C for 30 s, 95°C for 15 s, 60°C for 60 s, and melt for 15 s. The control *TIF-4A* was used for qPCR analysis of gene expression level to standardize the variance among the different templates (53, 57–59). Each detection was performed thrice. The cycle threshold (CT) values were converted to linear values using the comparative CT method and then analyzed with statistical algorithm geNorm (57). Student's *t*-tests were used to analyze the statistical data. All primer sequences are shown in Table S1.

### BmPGRP2 Localization, PGN Treatment, RNA Interference (RNAi), and BmPGRP2 Overexpression

A synthetic sequence includes *B. mori* A4 promoter (A4P) and GFP was cloned into the empty vector 1180 (GenBank: U13865.1) using SalI (#R3138V, NEB, USA) and BamHI (#R3136V, NEB, USA) restriction enzymes, followed adding *BmPGRP2-1* or *-2* [using BamHI and NotI (#R3189V, NEB, USA)] and Simian virus (SV)40 [using NotI and HindIII (#R3104V, NEB, USA)] to construct 1180-A4P-GFP-*BmPGRP2-1*/2-SV40, which was transfected into BmE cells for subcellular localization analysis. PGN-EB (PGN from *E. coli* 0111:B4, #tlrl-pgneb), PGN-BS (PGN from *Bacillus subtilis*, #tlrl-pgnbs), PGN-SA (PGN from *Staphylococcus aureus*, #tlrl-pgnsa), and LPS-EB (LPS from *E. coli* 0111:B4, #tlrl-eblps) were purchased from InvivoGen (San Diego, CA, USA) and added to BmE cells. Total RNA was extracted 0, 6, 12, and 24 h after treatment to detect the presence of *BmPGRP2-1, imd*, and the AMP gene *attacin* (*att*)*2*. Fifth instar silkworms were orally infected with *E. coli* and *S. marcescens* at 10^9^/larva, and extracted RNA was tested for the presence of *BmPGRP2-1/2* and the AMP gene *gloverin* (*glv*)*2*. Double-stranded (ds)RNA against *BmPGRP2-1* and dsRed were generated and added to BmN4-SID1 cells, whereas BmE cells were transfected with 1180 and 1180-A4P-*BmPGRP2-1*-SV40 (56). Total RNA was extracted to detect *BmPGRP2-1, imd, spz*, and *att2*. *BmPGRP2-2* RNAi and overexpression were carried out in a similar manner, and treated cells were then infected with BmNPV-GFP; DNA was extracted at 48 h post infection (hpi) for detection of copy number of BmNPV-GFP using control gene *GAPDH* and virus fluorescent was observed at 72 hpi (56).

### Protein Expression and Bond Test

The extracellular PGRP domain of BmPGRP2-1 was cloned with the primer BmPGRP2-1pro, which was used for prokaryotic expression in *E. coli* with the pSKB2-MsyB vector (pSKB2 vector was added with an MsyB tag in our lab). After induction of expression with 0.1 m mol/L isopropyl β-D-thiogalactoside (IPTG, #A100487, Sangon Biotech, China) at 16°C for 16 h, the protein in the supernatant was purified and used for western blotting with a BmPGRP2-1 antibody. Purified BmPGRP2-1 was tested for binding to PGN-EB, PGN-BS, and LPS-EB by enzyme-linked immunosorbent assay.

### Generation of Transgenic BmPGRP2-1 and-2 RNAi Silkworms

The transgenic *BmPGRP2-1* and *-2* RNAi vectors was constructed using *piggyBac* [3 × p3 EGFP afm] vector, respectively. The non-diapausing embryos of silkworm were used for microinjection. The G1 embryos were screened for transgenic silkworm. The insertion sites of transgenic silkworms were detected using inverse PCR analysis with the transposon-specific primers pBacL and pBacR (45, 55). There was only a single band after PCR amplification using pBacL and pBacR primers (data not shown), suggesting that there was inserted as a single copy (45, 55). Sequencing of the PCR products was blasted in SilkDB (http://silkworm.swu.edu.cn/silkdb/) will reveal the inserted region in silkworm genome. Fifth instar larvae and day 2 pupae of the transgenic PGRP2-1I and non-transgenic DZ strains were used for qPCR analysis of *BmPGRP2-1, imd, spz*, and *att2* expression. PGRP2-1I and DZ were orally infected with *E. coli* and *S. marcescens* at 10^9^/larva at the 5th instar; 2nd day pupae were injected with *S. marcescens* at 10/pupa. Third instar, 3rd instar molt, 4th instar, and 4th instar molt of the transgenic PGRP2-2I and non-transgenic 932 strains were examined for *BmPGRP2-2* expression by qPCR. Third instars of PGRP2-2I and 932 were orally infected with BmNPV at 3 × 10^5^ occlusion bodies (OB)/larva, and total DNA was extracted at 48 hpi (45, 55). RNA was extracted from PGRP2-2I and 932 at 0, 3, 6, 12, and 24 hpi for analysis of *BmPGRP2-2, imd, Relish, Myeloid differentiation primary response* (*MyD*)*88, Pelle*, and *AMP* gene expression.

### Transcriptome Analysis and Screening of Genes Downstream of BmPGRP2-2

PGRP2-2I and 932 were orally infected with BmNPV at 2.5 × 10^7^ OB/larva on day 3 of the 5th instar. RNA was extracted from the midgut and fat body at 3, 6, 12, and 24 hpi, and used for qPCR analysis of *BmPGRP2-2* expression. The RNA libraries of the 16 samples were constructed. Raw sequencing data were generated using an Illumina HiSeq 2000 system, which has been deposited in the NCBI, the BioProject ID is PRJNA521671. The polyA tails, noncoding RNAs and low-quality reads were removed from the raw reads to generate clean reads. The qualities of raw and clean reads were analyzed using FastQC (v0.11.1). Clean reads were mapped to the silkworm genome. The gene ontology (GO) analysis and KEGG annotation of the differentially expressed genes (DEGs) were executed using WEGO online and iPathCons, respectively (52, 60). *PTEN* was cloned using the BmPTEN ORF primer, and its expression after *BmPGRP2-2* RNAi and overexpression was evaluated. BmE cells were transfected with 1180 and 1180-A4P-*PTEN*-SV40; RNA was extracted and analyzed for *BmPGRP2-2* expression. The 1180-A4P-GFP-*PTEN*-SV40 vector was constructed and transfected into BmE cells for localization analysis.

### Analysis of Akt Phosphorylation and Cell Apoptosis

BmE cells were infected with BmNPV-GFP, and total protein was extracted for analysis of phosphorylated (p-)Akt expression at 0, 18, and 24 hpi. BmE cells were transfected with 1180 and the *BmPGRP2-2* overexpression vector. BmN4-SID1 cells were incubated with dsBmPGRP2-2 and dsRed, and treated cells were infected with BmNPV-GFP (56). GAPDH, p-Akt, and total Akt levels were assessed by western blotting. The antibody of GAPDH (#CB100127, California Bioscience, USA) was used as a control. The PVDF membrane (#03010040001, Roche, Switzerland) was blocked with 5% BSA for 12 h at 4°C after transfer film, followed incubation with antibody of p-Akt (Ser505, #4054, Cell Signaling Technology, USA) or total Akt (#9272, Cell Signaling Technology, USA) for 12 h at 4 °C. And then, the PVDF membrane was washed three times for 10 min each time with TBST, incubated with secondary antibody for 30 min at 37°C, washed 3 × 10 min with TBST, and treated with SuperSignal™ West Femto (#34095, Thermo Scientific, USA). The images were analyzed following the manufacturer's instructions. Cell apoptosis was detected at 24 hpi by flow cytometry. The cells were collected from each treatment, washed with cold PBS and incubated with Annexin V-fluorescein isothiocyanate (V-FITC) and propidium iodide (#K201-100, Biovision, USA) at 25°C for 20 min in the dark on ice. These samples were then analyzed by a fluorescence-activated cell sorter and Cell Quest software (BD, USA) following the manufacturer's instructions. Each test was repeated thrice. BmE cells were treated with the 10 μM PI3K inhibitor LY294002 (#HY-10108, MedChemExpress, USA), with dimethyl sulfoxide (DMSO, #D2650, Sigma, USA) treatment serving as a control (61). The expression of p-Akt was analyzed at 24 hpi and fluorescence was observed at 72 hpi.

## Results

### Cloning and Expression of BmPGRP2

**Figure 1:**
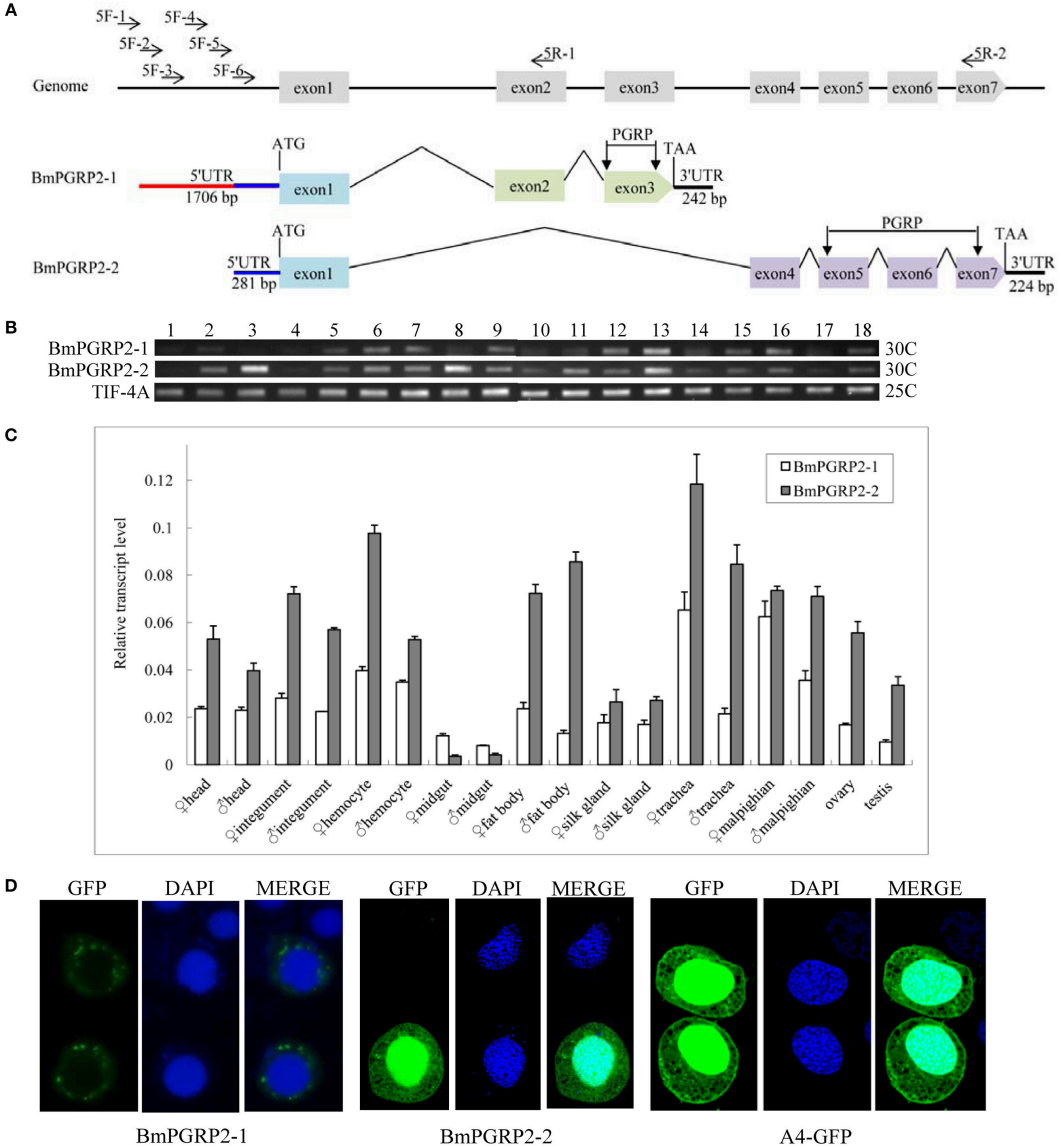
*BmPGRP2* expression patterns. **(A)** Schematic representation of *BmPGRP2* gene structure from DZ silkworm. Two forms of *BmPGRP2, BmPGRP2-1*, and *-2*, were cloned. The 5′ UTR was amplified with primers 5F-1,-2,-3,-4,-5, and-6 and 5R-1 and-2. The 5′ UTR of *BmPGRP2-1* was longer and contained that of *BmPGRP2-2*. **(B)** Analysis of *BmPGRP2* expression at whole individual of different developmental stages by RT-PCR. Points 1 to 4 represent 2-, 4-, 6-, and 8-day-old eggs; 5 to 13 represent hatched silkworm, first instar molt, second instar, second instar molt, third instar, third instar molt, fourth instar, fourth instar molt, and fifth instar larvae, respectively; 14 to 17 represent 2-, 4-, 6-, and 8-day-old pupae, respectively; and 18 represents moth. *TIF-4A* (GenBank: DQ443290.1) was the internal control. 25C and 30C represented the PCR amplification cycles was 25 and 30, respectively. **(C)** qPCR analysis of *BmPGRP2* expression in the head, cuticle, hemocyte, midgut, fat body, silk gland, trachea, malpighian, ovary, and testis of female and male silkworms. *TIF-4A* was used as the internal control. Bars represent standard deviation. **(D)** Subcellular localization of BmPGRP2. The vector 1180-A4P-GFP-*BmPGRP2-1/2*-SV40 and 1180-A4P-GFP-SV40 (A4-GFP) was transfected into BmE cells, respectively. The nucleus was dye blue by DAPI, and green fluorescence represented the location of target protein.

A bioinformatics analysis revealed that *BmPGRP2* exists as two forms, *BmPGRP2-1* and *-2*. *BmPGRP2-1* consisted of exons 1, 2, and 3 with a 242-bp 3′ UTR, whereas *BmPGRP2-2* consisted of exons 1, 4, 5, 6, and 7 with a 224-bp 3′ UTR (Figure 1A). To clone the 5′ UTR of *BmPGRP2-*1 and *-2*, we analyzed the 3000 bp upstream of the translation initiation site (ATG). There were four predicted transcription initiation sites (TIS) after analysis of promoter using online software (http://linux1.softberry.com/berry.phtml); forward primers (5F-1,-2,-3, and-4) were designed to target the 20–50 bp downstream of each candidate TIS. 5F-5 and 5F-6 were 300 and 600 bp downstream of 5F-4, respectively. The reverse primers 5R-1 and 5R-2 were located in exons 2 and 7, respectively (Figure 1A); 5R-1 and 5F-2,-3,-4,-5, and-6 amplified the target sequence but 5R-1 and 5F-1 did not amplify the target band; 5R-2 and 5F-6 amplified the target sequence but 5R-2 and 5F-1,-2,-3,-4, and-5 amplified none of the targets (data not shown). We confirmed by sequencing that the 1706-bp 5′ UTR of *BmPGRP2-1* containing the 281-bp 5′ UTR of *BmPGRP2-2* was cloned (Figure 1A). These results suggest that the two forms of *BmPGRP2* are generated from different TIS.

A sequence analysis suggested that BmPGRP2-1 and-2 contained a PGRP domain, while a phylogenetic analysis revealed that BmPGRP2-1 and-2 clustered into distinct classes (Figure S1). The RT-PCR results showed that *BmPGRP2-1* was more highly expressed in the larva than that in the egg (Figure 1B). The qPCR analysis showed that *BmPGRP2-1* and *-2* levels were lowest in the midgut, and that the expression of *BmPGRP2-2* was higher than that of *BmPGRP2-1* in all tissues of male and female larvae except for the midgut (Figure 1C). The predicted results from WoLF PSORT program on website showed that BmPGRP2-1 and-2 have no nucleotide localization sequences (NLS). A subcellular localization analysis indicated that BmPGRP2-1 was a transmembrane protein whereas BmPGRP2-2 was an intracellular protein (Figure 1D). The differences in phylogenetic position (Figure S1), temporal and spatial expression patterns (Figures 1B,C), and subcellular localization (Figure 1D) between the BmPGRP2-1 and-2 suggest that the two isoforms play distinct roles in host immune response to pathogens.

### BmPGRP2-1 Binds to a Dap-Type PGN and Activates Imd Signaling

**Figure 2:**
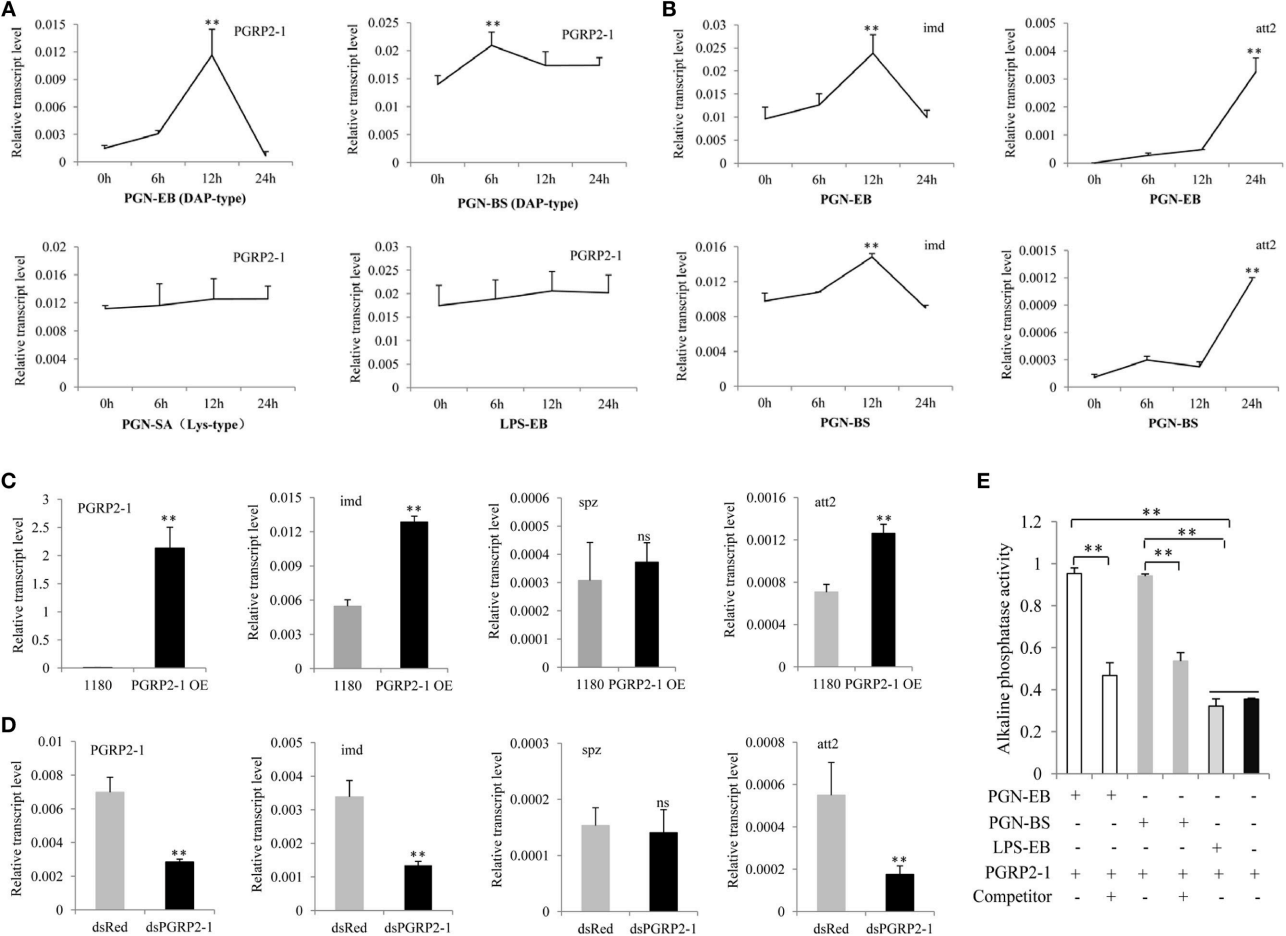
BmPGRP2-1 binds to DAP-type PGN and activates the Imd pathway. **(A)** qPCR analysis of *BmPGRP2-1* expression after PGN-EB (PGN from *E. coli* 0111:B4), -BS (PGN from *Bacillus subtilis*), and -SA (PGN from *Staphylococcus aureus*) and LPS-EB (LPS from *E. coli* 0111:B4) treatment. **(B)** Detection of *imd* and *att2* by qPCR in cells treated with PGN-EB and -BS. **(C)** *BmPGRP2-1* overexpression. The 1180-A4P-*BmPGRP2-1*-SV40 (PGRP2-1 OE) or empty 1180 vector was transfected into BmE cells. **(D)** RNAi of *BmPGRP2-1*. DsRNA targeting *BmPGRP2-1* (dsPGRP2-1) and control dsRed were added to BmN4-SID1 cells. *BmPGRP2-1, imd, spz*, and *att2* expressions were evaluated by qPCR. *TIF-4A* was used as the internal control of qPCR analysis, and each detection was performed thrice. **(E)** Interaction between BmPGRP2-1 and PGN-EB or -BS as determined by enzyme-linked immunosorbent assay. PGN-EB and -BS were set to each other's competitors; BmPGRP2-1 was pretreated with competitor (or left untreated) and the binding of BmPGRP2-1 with PGN-EB or -BS was evaluated based on alkaline phosphatase activity. LPS-EB was used as a control. Each assay was performed in triplicate. Student's *t*-tests were used to analyze the statistical data. ns: not significant. Bars represent standard deviation. **P < 0.01.

Multiple sequence alignment revealed that the zinc-binding cysteine residue of T7 lysozyme was replaced by a serine residue in BmPGRP2-1; the other seven PGRP proteins showed no amidase activity (Figure S2), suggesting that BmPGRP2-1 also lacks this activity. We tested whether BmPGRP2-1 can be induced by PGN. *BmPGRP2-1* and *glv2* but not *BmPGRP2-2* levels were induced in silkworm larvae by the G– bacteria *E. coli* and *S. marcescens* (Figure S3). A qPCR analysis showed that *BmPGRP2-1* expression was induced by DAP-type PGN-EB and PGN-BS but not LPS-EB or Lys-type PGN-SA (Figure 2A); *imd* and *att2* levels were also increased in BmE cells treated with PGN-EB and -BS (Figure 2B). However, Toll pathway activation in cells and individuals was unaltered by the treatment (data not shown). In addition, *BmPGRP2-1* overexpression in BmE cells increased the levels of *imd* and *att2* (Figure 2C), whereas silence of *BmPGRP2-1* had the opposite effect (Figure 2D) and *spz* level was unaltered. Thus, a DAP-type PGN induces BmPGRP2-1 to activate Imd signaling and AMP.

Some studies have reported that PGRP binds to PGN through the extracellular PGRP domain to activate Imd signaling (15, 24, 25). To determine whether BmPGRP2-1 can bind to a DAP-type PGN, we purified a prokaryotic protein with a PGRP domain (Figure S4A). Western blotting detected the target band in the purified protein (Figure S4B). Alkaline phosphatase activity was higher in BmPGRP2-1+PGN-EB and BmPGRP2-1+PGN-BS than in BmPGRP2-1+LPS-EB and BmPGRP2-1. In a competition assay using PGN-EB and PGN-BS, pretreatment of BmPGRP2-1 with a competitor decreased alkaline phosphatase activity (Figure 2E), demonstrating that BmPGRP2-1 directly binds to DAP-type PGN.

### BmPGRP2-1 Deficiency Decreases Resistance to Bacterial Infection in *B. mori*

**Figure 3:**
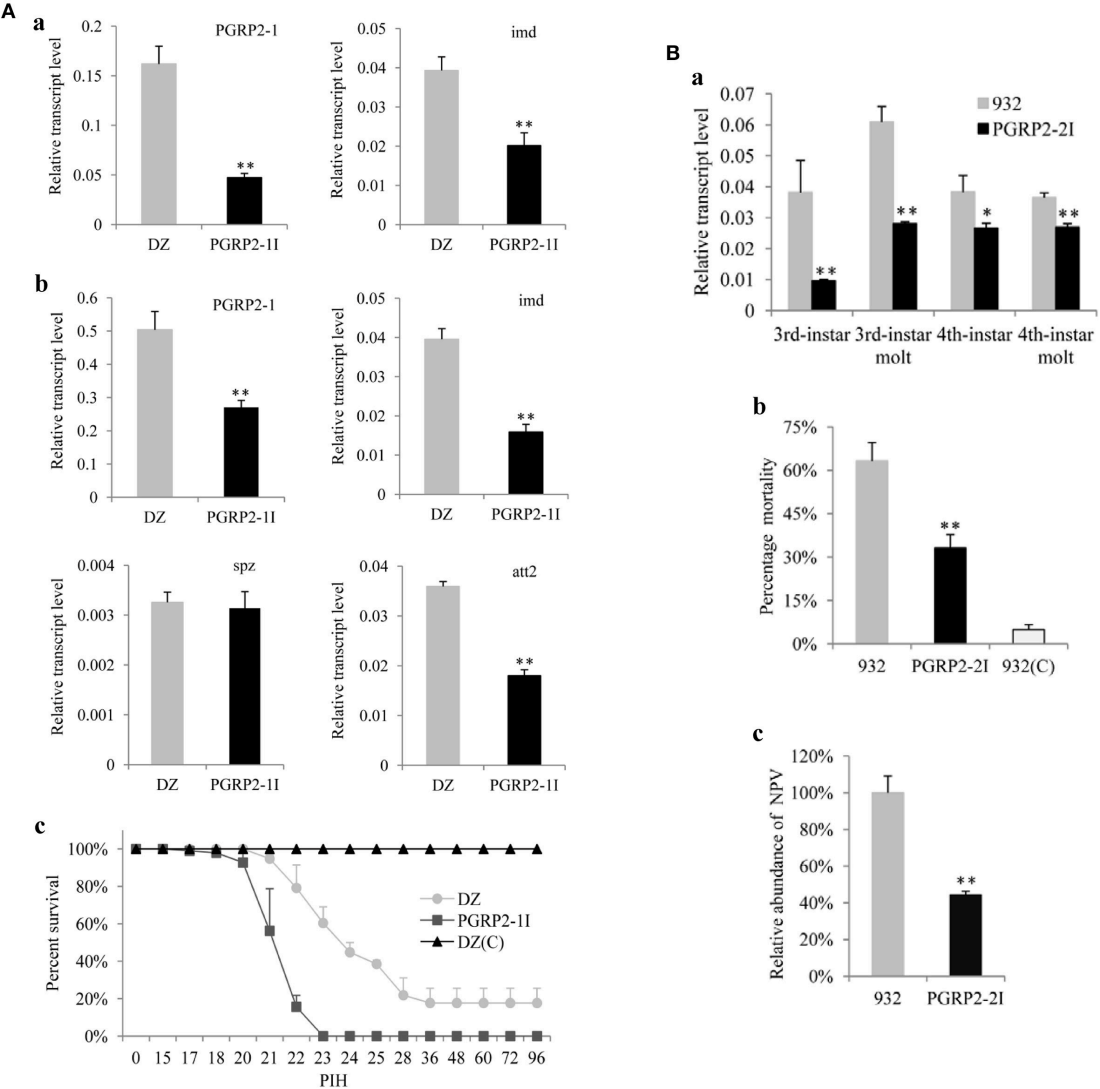
Analysis of transgenic RNAi silkworms. **(A)** Analysis of the transgenic line PGRP2-1I. Five larvae (a) and five pupae (b) were used to extract RNA for analysis of *BmPGRP2-1, imd, spz*, and *att2* expression by qPCR. *TIF-4A* was used as a control, and each assay was performed thrice. (c) Resistance analysis. Pupae of PGRP2-1I and DZ were injected with *S. marcescens* at 10/pupa. DZ(C) was the uninfected control. Each treatment had four repeats, and each repeat contained 25 pupae. PIH, post infection hour. **(B)** Analysis of PGRP2-2I. (a) *BmPGRP2-2* expression, as determined by qPCR. Each RNA sample was extracted from five larvae. *TIF-4A* was used as a control of qPCR, and each assay was performed thrice. (b) Mortality of *B. mori*. PGRP2-2I and 932 were orally infected at the 3rd instar stage with BmNPV using 3 × 10^5^ occlusion bodies (OB)/larva; 932(C) was the uninfected control. Each line contained triplicate replicates and each repeat had 70 larvae. (c) Analysis of viral DNA content by qPCR 48 h after BmNPV infection. Each DNA sample was extracted from 10 treated larvae. *GAPDH* (GenBank: AB262581.1) was used as the internal control, and each assay was performed thrice. Student's *t*-tests were used to analyze the statistical data. Bars represent standard deviation. *P < 0.05; **P < 0.01.

To further clarify the function of *BmPGRP2-1* in immune defense of silkworm against bacteria, we injected the transgenic RNAi vector pb-PGRP2-1I (Figure S5A) into embryos to generate a transgenic strain of PGRP2-1I (Figure S5B). The results of inverse PCR showed that a single copy of the transgene was detected in an intergenic region of the genome (Figure S5C). A qPCR analysis showed that *BmPGRP2-1* and *imd* levels were lower in PGRP2-1I as compared to DZ larvae (Figure 3Aa). However, the viability of PGRP2-1I and DZ strains was unaffected by oral infection with *E. coli* and *S. marcescens*. The expression of *BmPGRP2-1, imd*, and *att2* was also reduced in PGRP2-1I as compared to DZ pupae (Figure 3Ab), and the survival rate after *S. marcescens* injection was lower in PGRP2-1I than in DZ (Figure 3Ac). These results suggest that downregulation of *BmPGRP2-1* inhibits Imd signaling and reduces the resistance of silkworm to G– bacteria.

### BmPGRP2-2 Is Induced by BmNPV to Promote Viral Replication

**Figure 4:**
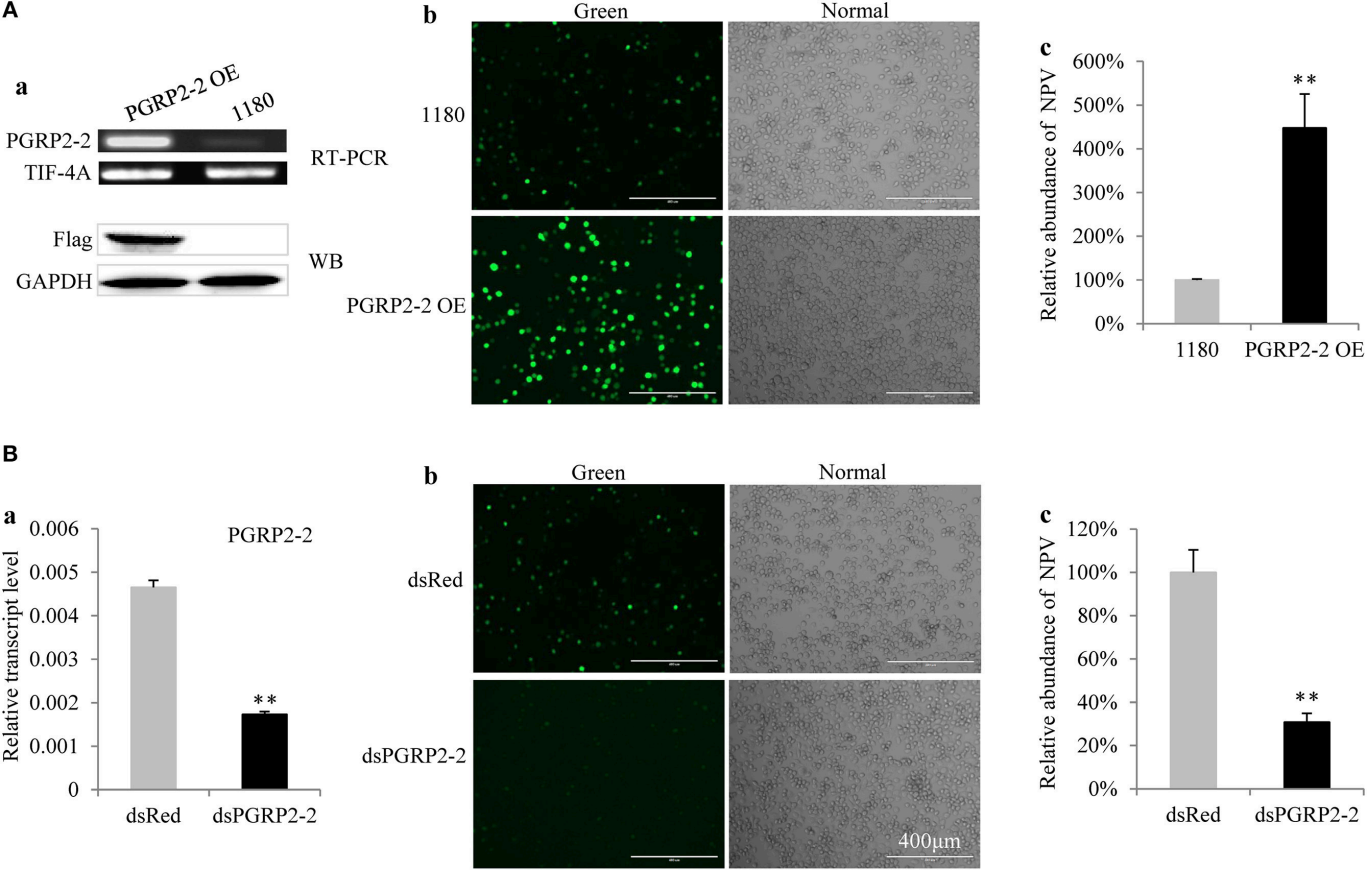
*BmPGRP2-2* regulates BmNPV multiplication. **(A)** *BmPGRP2-2* overexpression promoted BmNPV replication. A *BmPGRP2-2* overexpression vector with a flag tag (PGRP2-2 OE) was transfected into BmE cells; *BmPGRP2-2* transcript and protein were detected by RT-PCR (using control *TIF-4A*) and western blotting (using an anti-Flag antibody and control GAPDH antibody), respectively (a). The treated cells were infected with BmNPV-GFP. The green fluorescence of the virus was visualized at 72 hpi (b) and accumulated viral DNA content was detected by qPCR (using control *GAPDH*) at 48 hpi (c). **(B)** *BmPGRP2-2* knockdown inhibited BmNPV. The dsRNA of *BmPGRP2-2* (dsPGRP2-2) was added to BmN4-SID1 cells, and BmPGRP2-2 expression was determined by qPCR (using control *TIF-4A*) (a); viral green fluorescence (b) and BmNPV DNA content (using control *GAPDH*) were detected (c) after infection with BmNPV-GFP. Bars represent standard deviation. **P < 0.01.

*BmPGRP2-2* but not *BmPGRP2-1* expression was induced in BmE cells by BmNPV (Figure S6). To determine the role of *BmPGRP2-2* in BmNPV infection, BmE cells were transfected with a *BmPGRP2-2* overexpression vector containing a flag epitope. RT-PCR and western blot analyses confirmed that BmPGRP2-2 was upregulated in the cells (Figure 4Aa). Correspondingly, the fluorescence of the virus was enhanced (Figure 4Ab) and viral load was increased in cells overexpressing *BmPGRP2-2* relative to controls (Figure 4Ac). These results suggest that *BmPGRP2-2* overexpression promotes BmNPV multiplication. Furthermore, when *BmPGRP2-2* dsRNA was added to BmN4-SID1 cells, *BmPGRP2-2* expression was suppressed (Figure 4Ba), and viral fluorescence (Figure 4Bb) and viral DNA level (Figure 4Bc) were reduced, implying that *BmPGRP2-2* knockdown inhibits BmNPV replication. Together, these data demonstrated that BmNPV induces *BmPGRP2-2* to promote viral replication.

### Antiviral Effects of BmPGRP2-2 Are Not Mediated via Imd and Toll Pathways

To clarify the role of *BmPGRP2-2* in the immune response of silkworm larvae to BmNPV, we used the transgenic RNAi vector pb-PGRP2-2I (Figure S7A) and generated the transgenic 932 strains PGRP2-2I and PGRP2-2Ia (Figure S7B) containing the transgene insertion in an intergenic region and in the intron of a predicted gene, respectively (Figures S7C). PGRP2-2I was selected for subsequent analysis. *BmPGRP2-2* expression was decreased in PGRP2-2I as compared to the control 932 strain (Figure 3Ba). Moreover, mortality after BmNPV infection (Figure 3Bb) and accumulated BmNPV DNA content were significantly lower in PGRP2-2I than in 932 (Figure 3Bc). These results indicate that silencing *BmPGRP2-2* enhances the antiviral capacity of silkworm.

Since Imd and Toll signaling pathways are involved in the antiviral immune response (37–40), we investigated whether *BmPGRP2-2* activates these pathways. *BmPGRP2-2* was induced by BmNPV in larvae, with lower expression in PGRP2-2I than in 932 (Figure S8A). However, there was no difference in the levels of *imd, Relish, MyD88, Pelle, glv3*, and *glv4* of PGRP2-2I and 932 upon BmNPV infection (Figure S8B). These results suggest that *BmPGRP2-2* mediates its antiviral effects through a pathway other than canonical Imd and Toll signaling.

### BmPGRP2-2 Negatively Regulates BmPTEN

**Figure 5:**
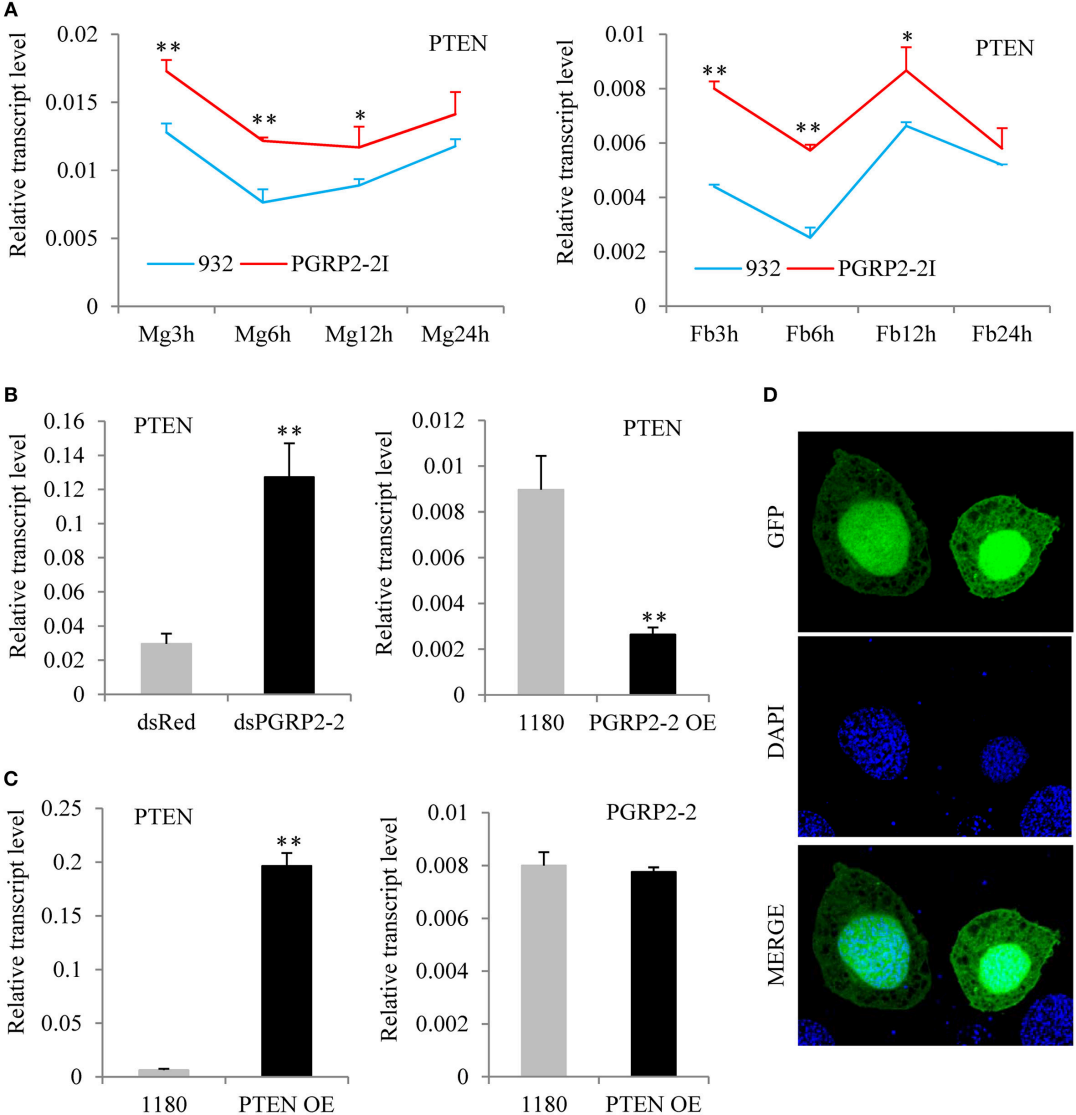
*BmPGRP2-2* negatively regulates *PTEN*. **(A)** qPCR analysis of *PTEN* expression (using control *TIF-4A*) in PGRP2-2I. PGRP2-2I and 932 were orally infected with BmNPV on day 3 of the 5th instar. RNA was extracted from the midgut (MG) and fat body (Fb) at 3, 6, 12, and 24 hpi. **(B)** Detection of *PTEN* (using control *TIF-4A*) after *BmPGRP2-2* RNAi and overexpression. **(C)** qPCR analysis of *BmPGRP2-2* (using control *TIF-4A*) after *PTEN* overexpression **(D)** Subcellular localization of PTEN. The vector 1180-A4P-GFP-*PTEN*-SV40 was transfected into BmE cells. The green fluorescence represented the location of target protein and blue indicated nucleus. Bars, standard deviations. Significant differences, **P < 0.01, *P < 0.05.

To identify the signaling pathway downstream of *BmPGRP2-2*, RNA was extracted from the midgut and fat body of PGRP2-2I and 932 at 3, 6, 12, and 24 hpi (Figure S9A). We confirmed that *BmPGRP2-2* expression in both tissues was significantly lower in PGRP2-2I than in 932 by qPCR (Figure S9B). And then, the 16 RNA samples were used for RNA-seq (Supplementary File 1). The FPKM values of silkworm immune-related genes were shown in Table S2 after information analysis. The transcriptome analysis revealed that *BmPTEN* expression was higher in the midgut and fat body of PGRP2-2I than in those of 932 (Figure S10); this was confirmed by qPCR (Figure 5A). We also found that *BmPGRP2-2* knockdown and overexpression increased and decreased the expression level of *BmPTEN*, respectively (Figure 5B), indicating a negative regulatory relationship. We also examined whether *BmPGRP2-2* is regulated by *BmPTEN*; however, *BmPTEN* overexpression did not affect *BmPGRP2-2* expression (Figure 5C). BmPTEN was an intracellular protein (Figure 5D). These results indicate that *BmPTEN* is downstream of and negatively regulated by *BmPGRP2-2*.

### BmPGRP2-2 Regulates p-Akt and Cell Apoptosis

**Figure 6:**
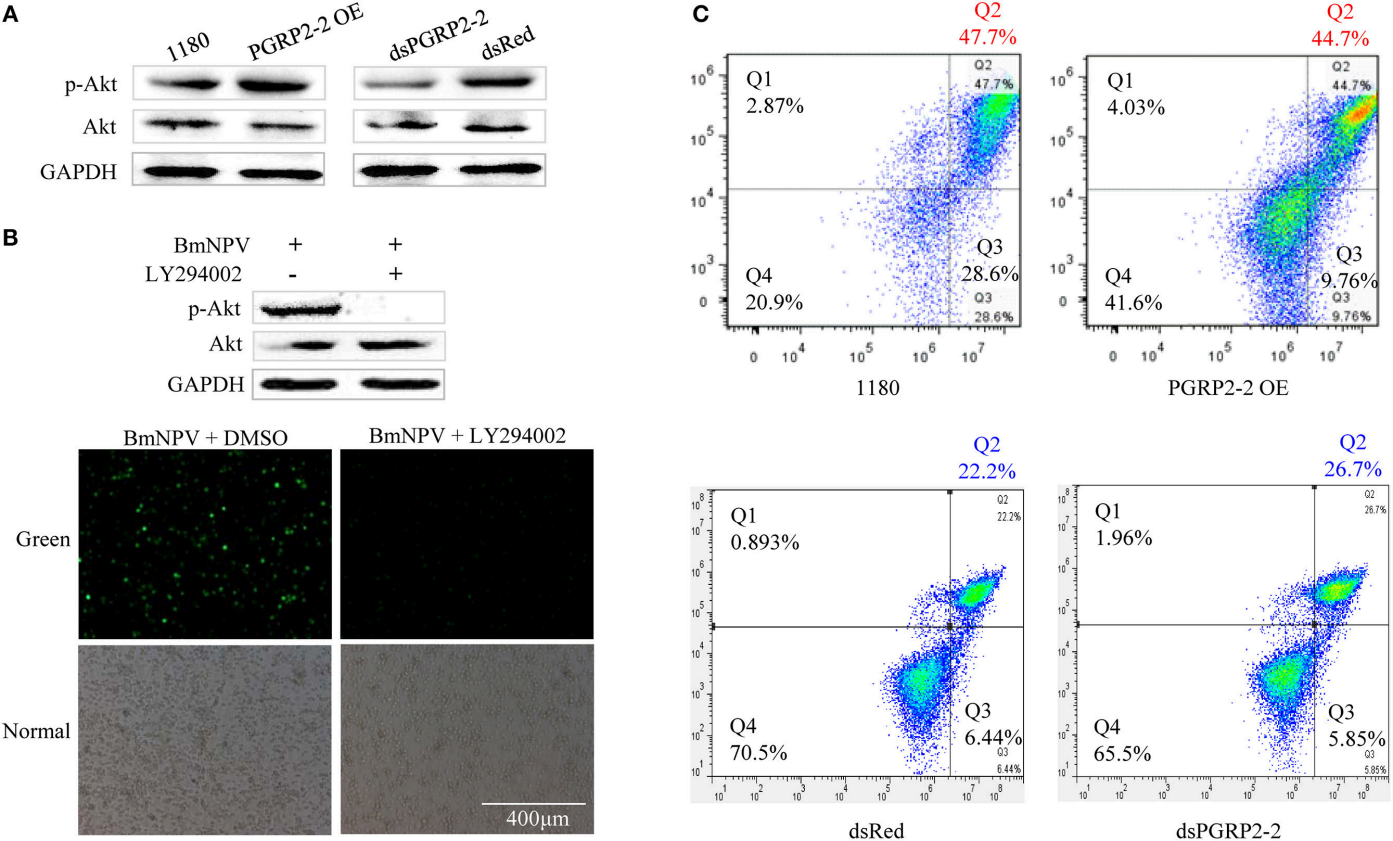
*BmPGRP2-2* regulates Akt phosphorylation and cell apoptosis. **(A)** Detection of p-Akt by western blotting. Following *BmPGRP2-2* RNAi and overexpression, cells were infected with BmNPV-GFP; protein was extracted at 24 hpi. **(B)** Treatment with the PI3K inhibitor LY294002 (10 μM) inhibits Akt phosphorylation and BmNPV replication. BmE cells were treated with BmNPV-GFP+LY294002; BmNPV-GFP+dimethyl sulfoxide (DMSO) was used as a control. Akt phosphorylation was evaluated at 24 hpi, and fluorescence associated with the virus was detected. **(C)** Analysis of cell apoptosis. Following *BmPGRP2-2* RNAi and overexpression, apoptotic cells were detected by flow cytometry at 24 hpi. X and Y axis represented Annexin V-fluorescein isothiocyanate (V-FITC) and propidium iodide (PI), respectively. Late apoptotic, early apoptotic, and non-apoptotic cells were assigned to Q2, Q3, and Q4 section, respectively.

*Autographa californica* multiple nucleopolyhedrovirus (AcMNPV) infection increases Akt phosphorylation in Sf9 cells (61). Akt is a downstream effector of PI3K-dependent cell survival, whereas PTEN is an inhibitor of PI3K/Akt signaling (62, 63). We therefore investigated whether p-Akt is upregulated in BmE cells after BmNPV infection. BmNPV induced Akt phosphorylation, with the maximum level detected at 24 hpi (Figure S11A). We also examined found that *BmPGRP2-2* overexpression and knockdown increased and decreased Akt phosphorylation, respectively, at 24 hpi (Figure 6A). Moreover, pharmacological inhibition of p-Akt by treatment with the PI3K inhibitor LY294002 suppressed BmNPV replication (Figure 6B).

Previous studied have shown that Akt plays an important role in promoting cell survival by suppressing apoptosis (63, 64). We examined whether *BmPGRP2-2* regulates apoptosis via modulation of Akt activation. BmNPV infection of BmE cells resulted in the formation of apoptotic bodies (Figure S11B). A flow cytometry analysis showed that *BmPGRP2-2* overexpression and knockdown reduced and increased the apoptotic fraction, respectively (Figure 6C). These results indicate that *BmPGRP2-2* induced by BmNPV inhibits apoptosis via Akt activation.

## Discussion

PGRP is well-known to play an important role in immune defense of insects against bacteria. In this study, we demonstrated that BmPGRP2-1 binds to DAP-type PGN to activate Imd signaling and inhibit bacteria, whereas BmNPV-induced *BmPGRP2-2* suppresses host cell apoptosis to enable viral replication.

Multiple splice forms of PGRP-L have been identified in *Drosophila* and *A. gambiae*, although it is unclear how these are generated (5, 12, 15, 16). Our results indicate that the two forms of *BmPGRP2* are transcribed from different TIS. *BmPGRP2-1* was generated from the distal TIS and contained a long 5′ UTR, whereas *BmPGRP2-2* with a short 5′ UTR was transcribed from the proximal TIS. BmNPV is the primary pathogen to silkworm (46); we speculate that *BmPGRP2-1* is the evolutionarily more ancient form, and that *BmPGRP2-2* arose through interactions between the silkworm and BmNPV.

The two forms of *BmPGRP2* showed distinct expression patterns, subcellular localization, and roles in the immune response to different pathogens. BmPGRP2-1 is a transmembrane protein that binds to DAP-type PGN via the extracellular PGRP domain, thereby activating the Imd pathway and AMP, similar to PGRP-LC in *Drosophila*. We therefore speculate that BmPGRP2-1 interacts with Imd to activate downstream signaling molecules through an PGRP-LC-like intracellular domain (15, 24, 25). Previous studies have demonstrated that DAP-type PGNs activates Imd signaling, whereas Lys-type PGNs activate the Toll pathway (8, 65). We found here that BmPGRP2-1 was induced by G– bacteria and that silencing BmPGRP2-1 expression suppressed Imd signaling and AMP and reduced the resistance of transgenic silkworm to G– bacteria, confirming that BmPGRP2-1 plays a key role in the immune defense against G– bacteria and possibly against some G+ bacteria with DAP-type PGNs, although the latter requires confirmation.

*BmPGRP2-2* was induced by BmNPV to promote viral replication; on the other hand, *BmPGRP2-2* knockdown inhibited BmNPV. Some studies have reported that the Imd and Toll pathways are involved in antiviral immune response; inhibition of Imd signaling increased CrPV and alphavirus replication in *Drosophila* (37, 38) whereas Toll pathway activation suppressed dengue virus replication in *A. aegypti* (40). The expression of PGRP-LA and PGRP-SC1A was up-regulated in *Drosophila* after infection of Nora virus (66). Sigma virus infection induced the expression of PGRP-SB1, PGRP-SD, and some AMP genes without altering Toll and Imd signaling in *Drosophila* (67). Imd pathway activation inhibited BmCPV in silkworm (53). The results of the present study demonstrate that antiviral signaling of *BmPGRP2-2* against BmNPV is independent of the two pathways. The results of our previous study (53) and present study revealed the different responses of Imd pathway to different viruses in silkworm. JAK/STAT pathway could be activated by BmNPV but not BmCPV (34). This was unexpected, given that *BmPGRP2-2* was found to negatively regulate PTEN and activate Akt to inhibit apoptosis. PGRP-LB has been shown to function as an negative regulator of Imd pathways in *Drosophila* (26). It was previously reported that PI3K-Akt signaling is required for efficient Baculovirus replication (61). The Hepatitis B virus HBx protein suppressed PTEN expression and activated Akt phosphorylation (68), while the hepatitis C virus NS5A protein-induced suppression of PTEN abrogated its inhibitory effect on PI3K/Akt signaling, triggering Akt activation to promote cell survival (69). This study demonstrated that virus-induced PRRs in the host negatively regulate the PTEN-PI3K/Akt pathway. Thus, the mechanism of BmNPV infection is similar to those of some human viruses, suggesting that *B. mori* and BmNPV can serve as a model of human–virus interaction, for instance to identify new host genes that are involved in viral infection and to screen antiviral drugs targeting the PTEN-PI3K/Akt pathway.

**Figure 7:**
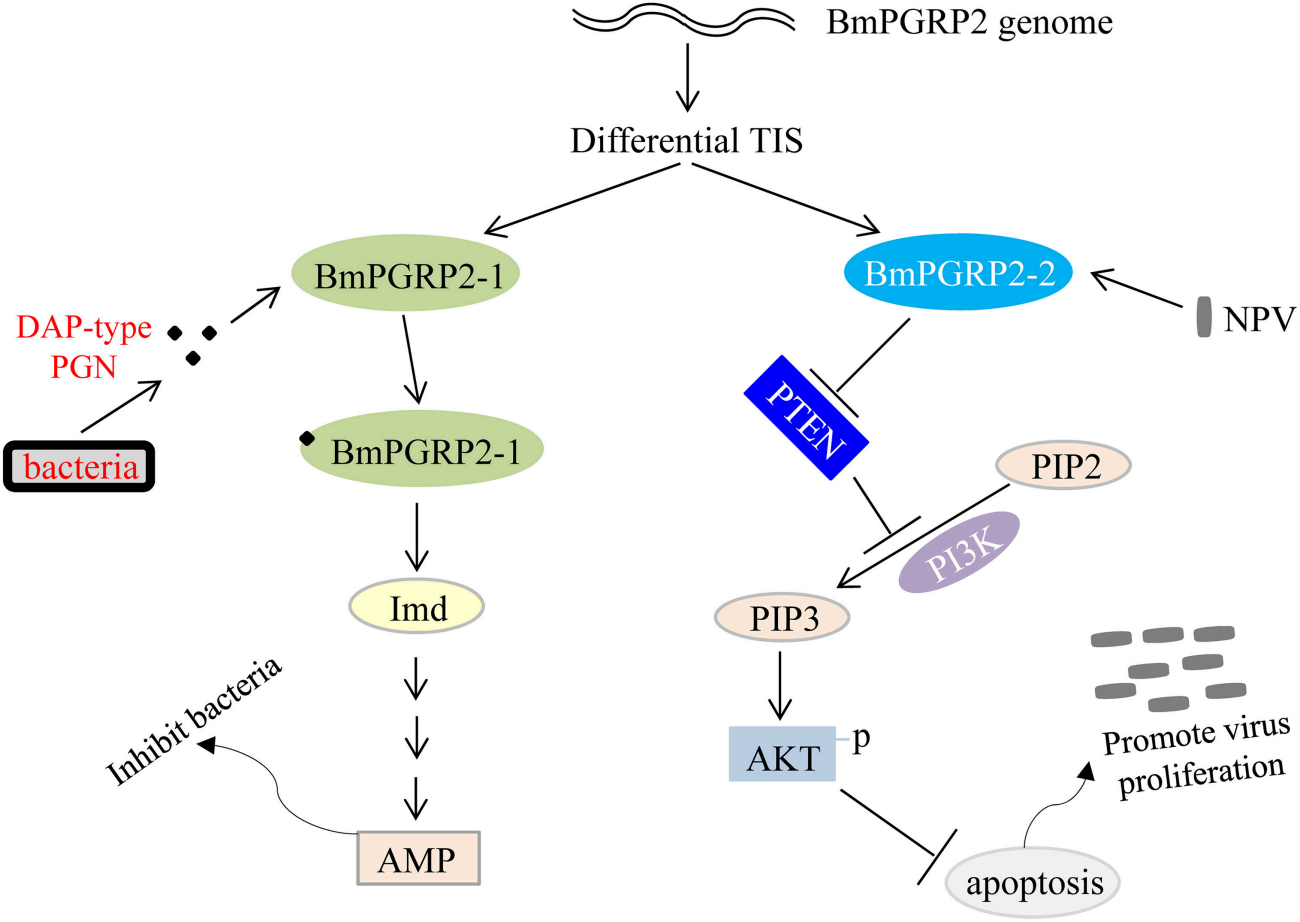
Model of *BmPGRP2* functional differentiation in host immune response to pathogens. The two isoforms of *BmPGRP2* were generated from different transcription initiation sites (TIS). BmPGRP2-1 recognizes and binds to DAP-type PGN through the extracellular PGRP domain and activates the Imd pathway and AMP to inhibit bacteria; *BmPGRP2-2* is induced by BmNPV to suppress *PTEN*, thereby relieving its inhibition of PI3K/Akt signaling and triggering an increase in Akt phosphorylation and activation to inhibit cell apoptosis. The increased cell survival is beneficial for viral replication. (Part of the evidence for protein levels is lacking).

We found that *BmPGRP2-2* negatively regulates PTEN-PI3K/Akt signaling to inhibit cell apoptosis, in contrast to the classic Imd and Toll pathways that are activated by PGRP. Apoptosis plays a key role in host defense against viral infection; however, viruses use various strategies to support cell survival, which is in turn beneficial for viral propagation (69). *BmPGRP2-1* was induced by G– bacteria to activate Imd signaling and inhibit bacterial proliferation, whereas *BmPGRP2-2* was induced by BmNPV to promote cell survival and consequently, viral replication. Thus, PGRP not only mediates host immune defense against bacteria but is also used by viruses to evade host antiviral defense systems (Figure 7). Some open questions are whether *BmPGRP2-2* and other PRRs are used by other viruses; which viral components are recognized by BmPGRP2-2; the mechanism underlying negative regulation of PTEN by *BmPGRP2-2*; and whether there are other host genes or immune components involved in BmPGRP2-PTEN-PI3K/Akt signaling.

Some drugs targeting the PTEN-PI3K/Akt pathway can inhibit viral infection. Blocking virus-induced PI3K/Akt signaling inhibited the replication of influenza virus (70). Emodin suppressed p-Akt to induce hepatocellular carcinoma cell apoptosis (71), whereas geridonin combined with paclitaxel induced apoptosis and inhibited the proliferation of gastric cancer cells via upregulation of PTEN and suppression of Akt phosphorylation (72). The PI3K inhibitor LY294002 was shown to block Akt activation and thereby inhibit AcMNPV (61) and BmNPV (this study). These drugs may be used in sericulture to control BmNPV infection if the production process and purity can be optimized. We have generated transgenic antiviral silkworms that block BmNPV infection via overexpression of *Bmlipase-1* (55); by suppressing BmNPV mRNA by viral gene RNAi (45); and by inhibiting BmNPV protein synthesis via *hycu-ep32* overexpression (73). In the present study, we confirmed that modulating host immune defense can inhibit BmNPV; overexpressing antiviral genes and dsRNA targeting viral genes can further enhance host resistance (74). Hence, combining four antiviral strategies (*Bmlipase-1* and *hycu-ep32* overexpression and silencing of *BmPGRP2-2* and various viral genes) can potentially yield a transgenic silkworm with high resistance to BmNPV.

In conclusion, we cloned two forms of *BmPGRP2* generated from different TIS and characterized their distinct functions in the host immune response to pathogens (Figure 7). Our findings indicate that *BmPGRP2-2* does not function in canonical immune signaling pathways and PGRP2 is not only involved in host immune defense against invading pathogens, but is also used by viruses to evade host antiviral mechanisms.

## Author Contributions

LJ and QX designed research. LJ, HG, TC, WY, and QX analyzed data. LJ, WL, YD, QS, BW, YW, and EX performed experiments. LJ and QX wrote the manuscript.

### Conflict of Interest Statement

The authors declare that the research was conducted in the absence of any commercial or financial relationships that could be construed as a potential conflict of interest.

## Abbreviations

PRRs: pattern-recognition receptors
PAMPs: pathogen-associated molecular patterns
LPS: lipopolysaccharides
PGRP: peptidoglycan recognition protein
PGRP-S: short PGRPs
PGRP-L: long PGRPs
PGNs: peptidoglycans
DAP-type: diaminopimelic acid type
Lys-typ: Lysine typ
Imd: immune deficiency
G–: gram-negative
G+: gram-positive
AMPs: antimicrobial peptides
GNBPs: G–
binding proteins: TLRs: Toll-like receptors
proPO: prophenoloxidase
Spz: Spatzle
*E. coli*: *Escherichia coli*
*S. marcescens*: *Serratia marcescens*
CrPV: cricket paralysis virus
*B. mori*: *Bombyx mori*
BmNPV: *B. mori* nucleopolyhedrovirus
BmCPV: *B. mori* cytoplasmic polyhedrosis virus
AcMNPV: *Autographa californica* multiple nucleopolyhedrovirus
PTEN: phosphatase and tensin homolog
PI3K: phosphoinositide 3-kinase
DZ: Dazao
BmNPV-GFP: BmNPV expressing green fluorescent protein
RT-PCR: reverse transcription PCR
qPCR: quantitative PCR
ORF: open reading frame
UTR: untranslated region
CT: cycle threshold
RNAi: RNA interference
A4P: A4 promoter
SV40: Simian virus 40
PGN-EB: PGN from *E. coli* 0111:B4
PGN-BS: PGN from *Bacillus subtilis*
PGN-SA: PGN from *Staphylococcus aureus*
LPS-EB: LPS from *E. coli* 0111:B4
*att2*: *attacin 2*
*glv2*: *gloverin 2*
dsRNA: double-stranded RNA
hpi: h post infection
pSKB2-MsyB: pSKB2 vector with an MsyB tag
*MyD88*: *Myeloid differentiation primary response 88*
OB: occlusion bodies
GO: gene ontology
DEGs: differentially expressed genes
p-Akt: phosphorylated Akt
V-FITC: V-fluorescein isothiocyanate
IPTG: isopropyl β-D-thiogalactoside
DMSO: dimethyl sulfoxide
ATG: translation initiation site
TIS: transcription initiation sites
NLS: nucleotide localization sequences.
