# Supplementary material for: Distinct Functions of Bombyx mori Peptidoglycan Recognition Protein 2 in Immune Responses to Bacteria and Viruses
Source: Front Immunol. 2019 Apr 12;10:776. doi: 10.3389/fimmu.2019.00776 (PMC6473039; doi:10.3389/fimmu.2019.00776)
Supplement: Supplementary file 1 [file Data_Sheet_1.docx]

**
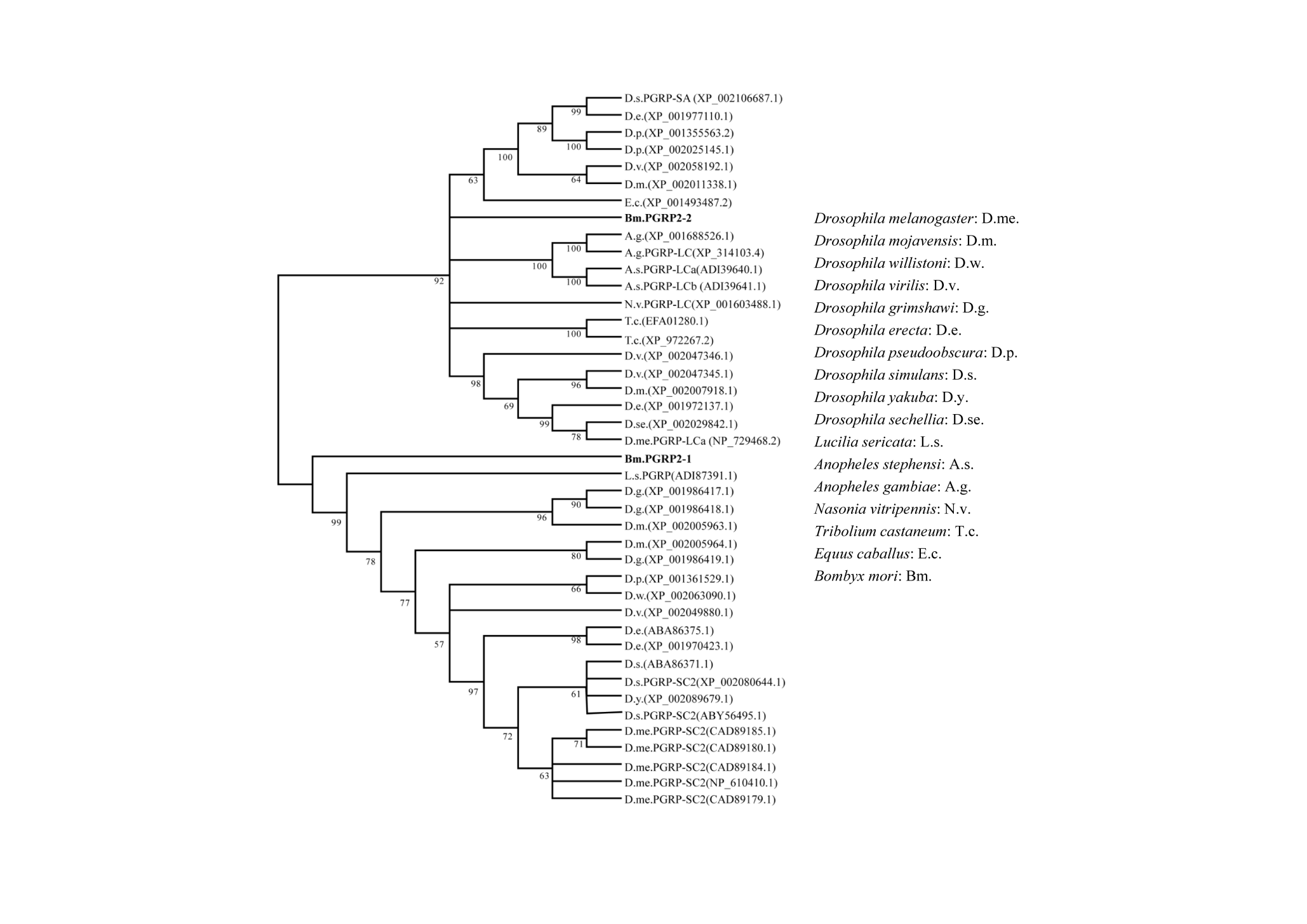
**

**Figure S1.** Phylogenetic analysis of *BmPGRP2*. Sequences homologous to *BmPGRP2-1* and *-2* were searched in NCBI. The 20 mostly similar sequences of each were used for phylogenetic analysis with MEGA. The gene ID is shown in the brackets.

**
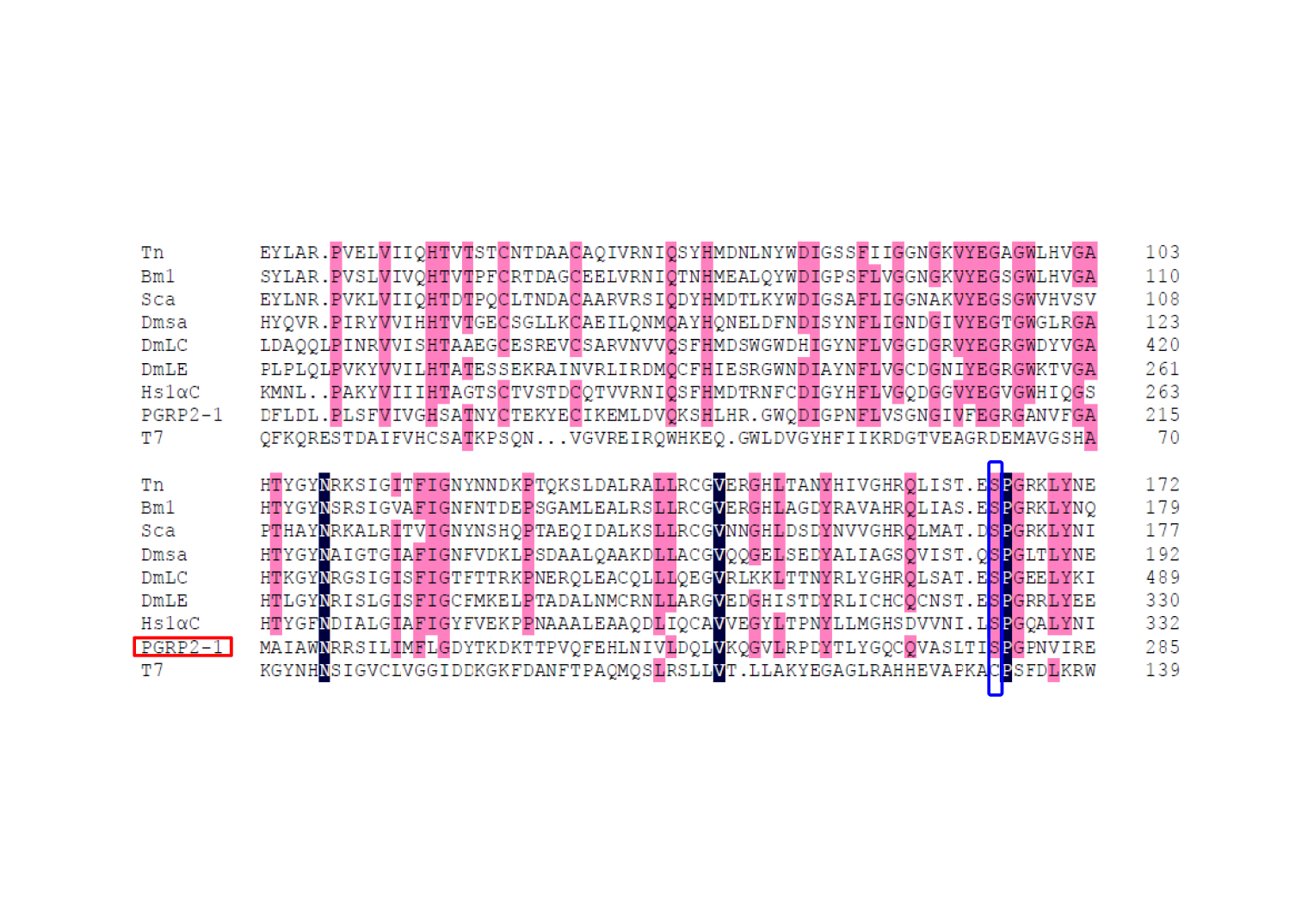
**

**Figure S2.** Multiple sequence alignment of *BmPGRP2-1*. Amino acid sequences of PGRPs of *Trichoplusia ni* (Tn, AAC31820), *B. mori* (Bm1, BAA77209), *Samia cynthia* (Sca, BAF03522), *D. melanogaster* (PGRP-SA, DmSA; PGRP-LC, DmLC; and PGRP-LE, DmLE), *Homo sapiens* (HsIɑC, AAK72484), and T7 lysozyme (T7, NP_0419731) were downloaded from NCBI, which were used for multiple sequence alignment with BmPGRP2-1 using ClustalX soft. The blue box indicated that the amidase active site of T7 lysozyme. BmPGRP2-1 has no amidase activity.


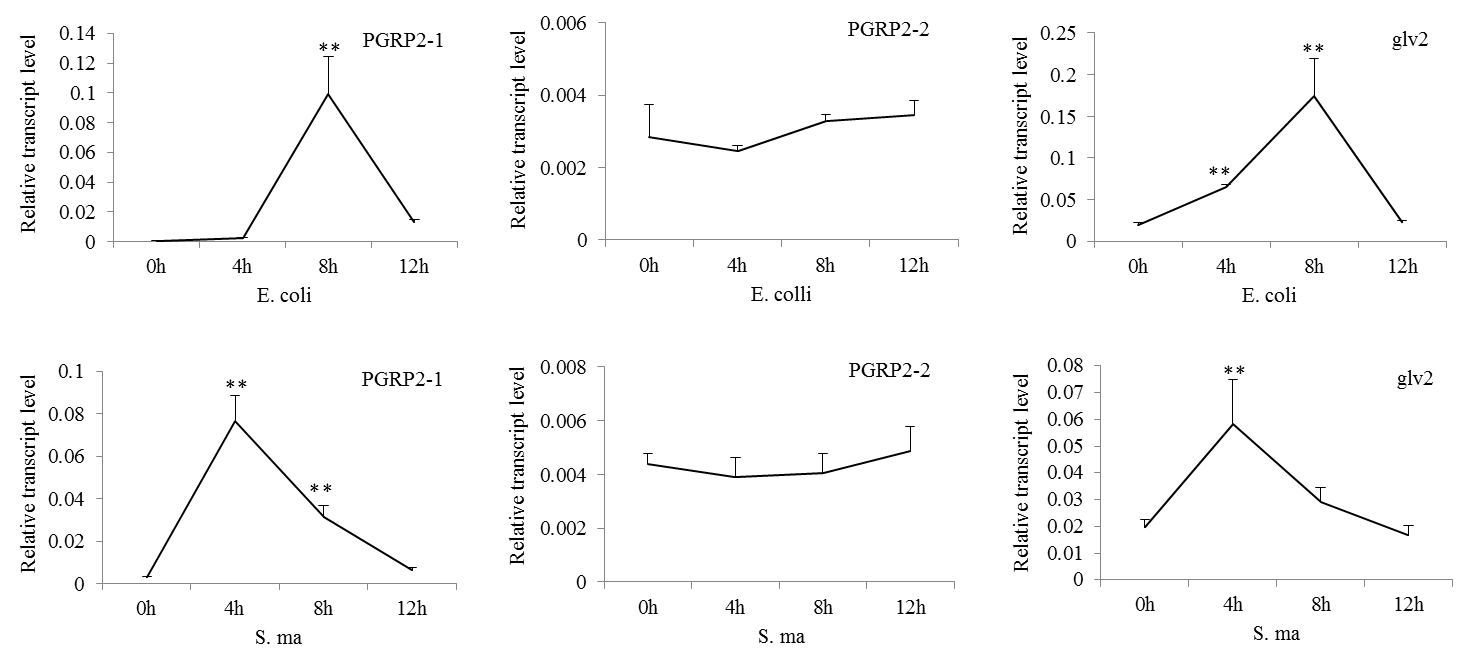


**Figure S3.** qPCR analysis of *BmPGRP2-1/2* and *glv2* in 5th instar silkworms orally infected with *E. coli* (10^9^/larva) and *S. marcescens* (10^9^/larva). Five treated larvae were used for RNA extraction. *TIF-4A* was used as a control, and each assay was performed thrice. Student’s *t*-tests were used to analyze the statistical data. Bars represent standard deviation. ***P* < 0.01.


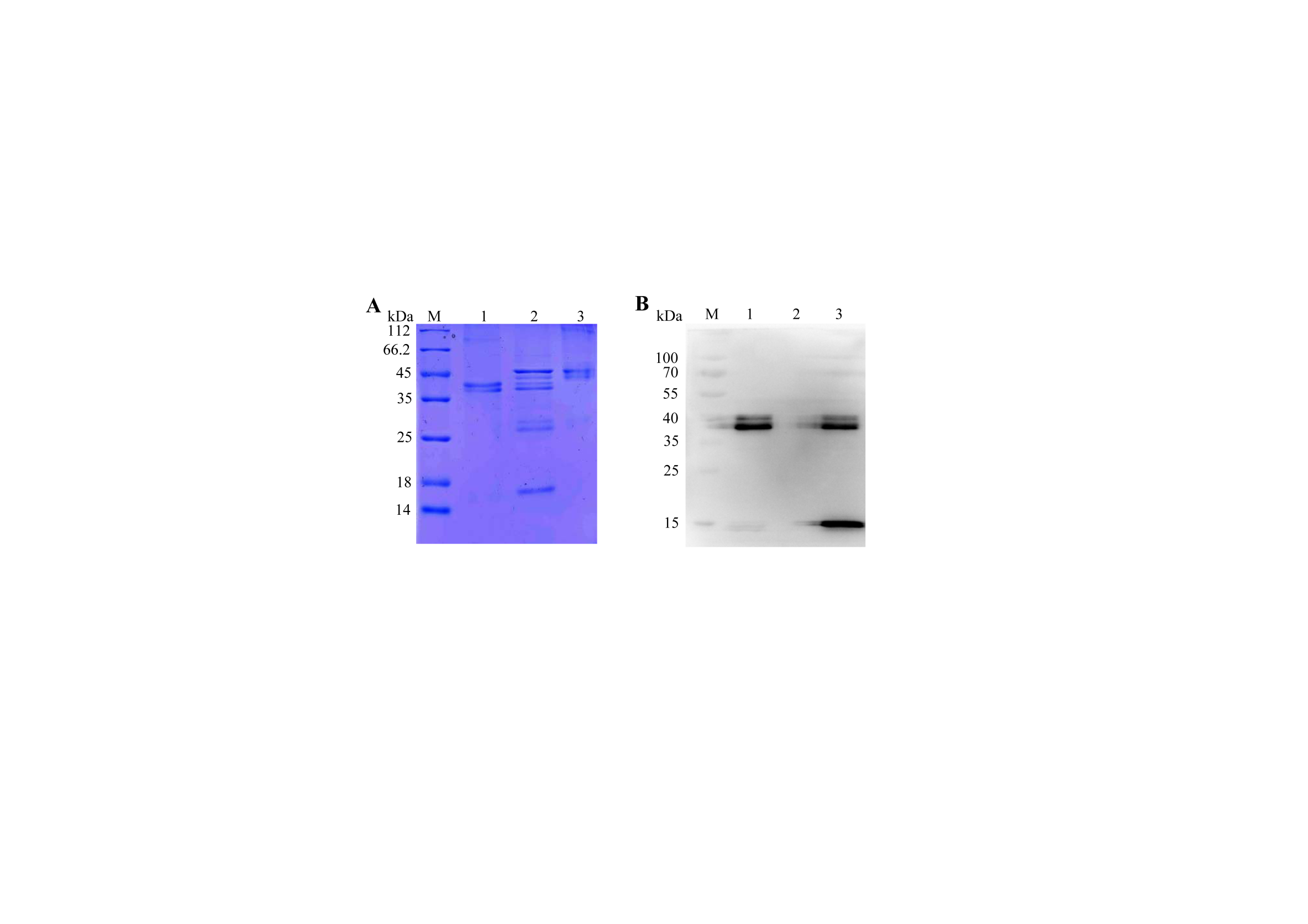


**Figure S4.** Detection of purified BmPGRP2-1. The extracellular PGRP domain of BmPGRP2-1 in the pSKB2-MsyB vector (pSKB2 vector was added with an MsyB tag in our lab) was used for prokaryotic expression. (A) Detection of digestion products. Points 1 to 3 represent the BmPGRP2-1/MsyB fusion protein, digestion products, and protease used for digestion, respectively. (B) Western blotting analysis with BmPGRP2-1 antibody. Points 1 to 3 represent the BmPGRP2-1/MsyB fusion protein, protease for used for digestion, and digestion products, respectively. M, marker.


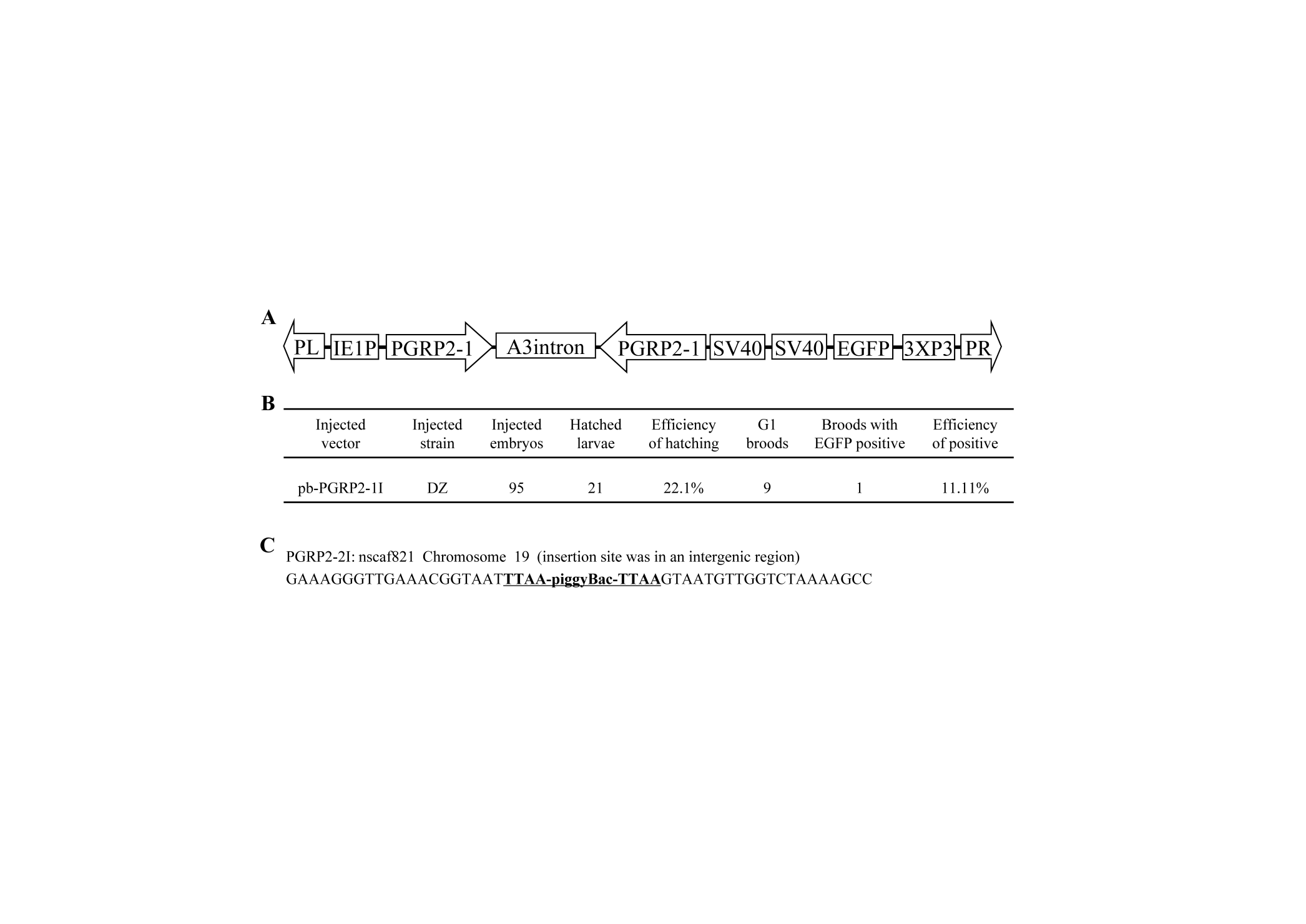


**Figure S5.** Construction of transgenic BmPGRP2-1 RNAi silkworm. (A) Schematic illustration of the transgenic RNAi vector. (B) Results of embryo microinjection. (C) Analysis of insertion site using inverse PCR with the transposon-specific primers pBacL and pBacR [[45](#_ENREF_45), [55](#_ENREF_55)].


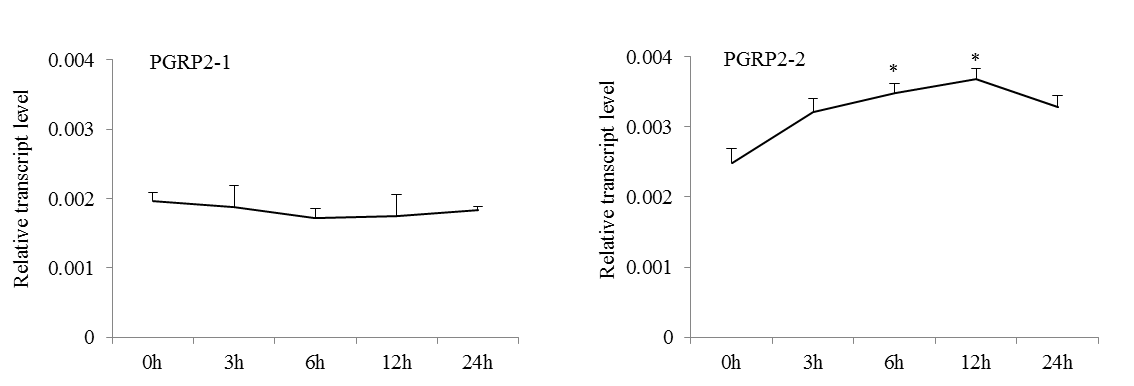


**Figure S6.** qPCR analysis of *BmPGRP2* expression in BmE cells following BmNPV treatment. BmE cells were infected with BmNPV at multiplicity of infection of 1 (MOI 1), the RNA was extracted at 0, 3, 6, 12, and 24 hpi for qPCR analysis of *BmPGRP2-1* and *-2*. *TIF-4A* was used as a control, and each assay was performed thrice. Student’s *t*-tests were used to analyze the statistical data. Bars represent standard deviation. Bars represent standard deviation. **P* < 0.05.


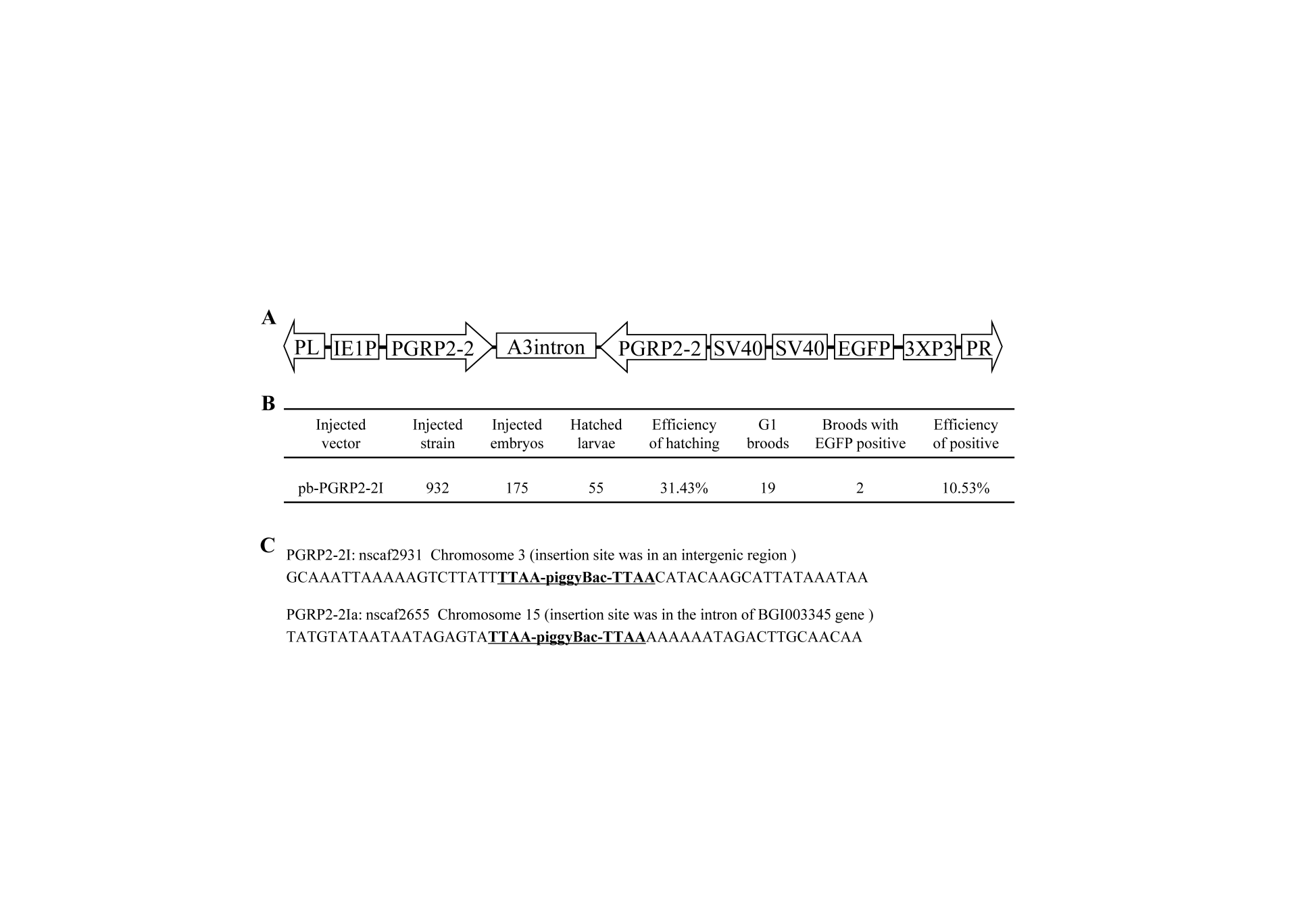


**Figure S7.** Construction of transgenic BmPGRP2-2 RNAi silkworm. (A) Schematic illustration of transgenic RNAi vector. (B) Results of embryo microinjection. (C) Detection of insertion sites using inverse PCR with the transposon-specific primers pBacL and pBacR [[45](#_ENREF_45), [55](#_ENREF_55)].


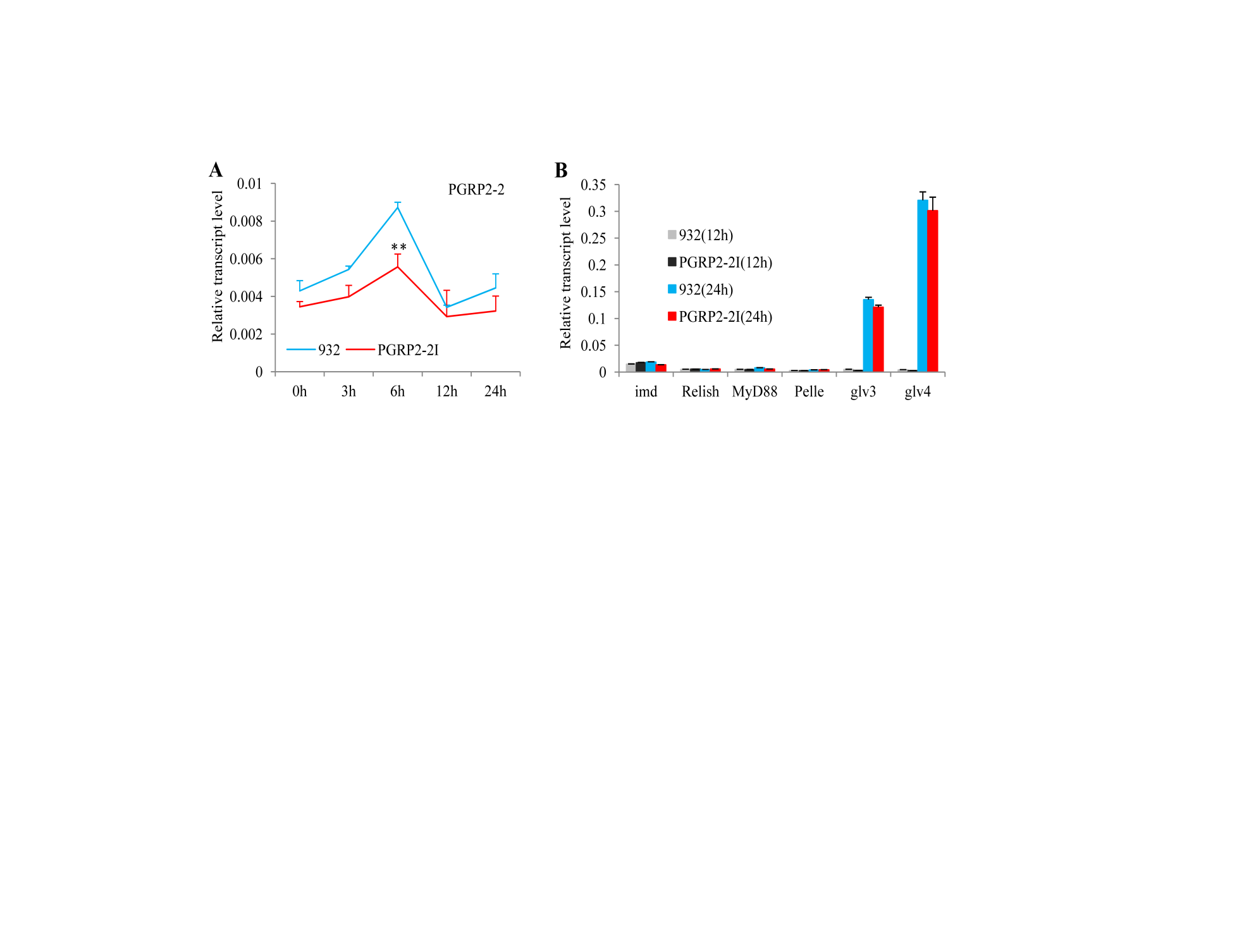


**Figure S8.** Analysis of Imd and Toll pathways components by qPCR. The 3rd instar of PGRP2-2I and 932 were orally infected with BmNPV; RNA was extracted at 0, 3, 6, 12, and 24 hpi. (A) qPCR analysis of *BmPGRP2-2* expression. (B) Detection of *imd*, *Relish*, *MyD88*, *Pelle*, *glv3*, and *glv4* by qPCR. *TIF-4A* was used as a control, and each assay was performed thrice. Student’s *t*-tests were used to analyze the statistical data. Bars represent standard deviation. Bars represent standard deviation. ***P* < 0.01.


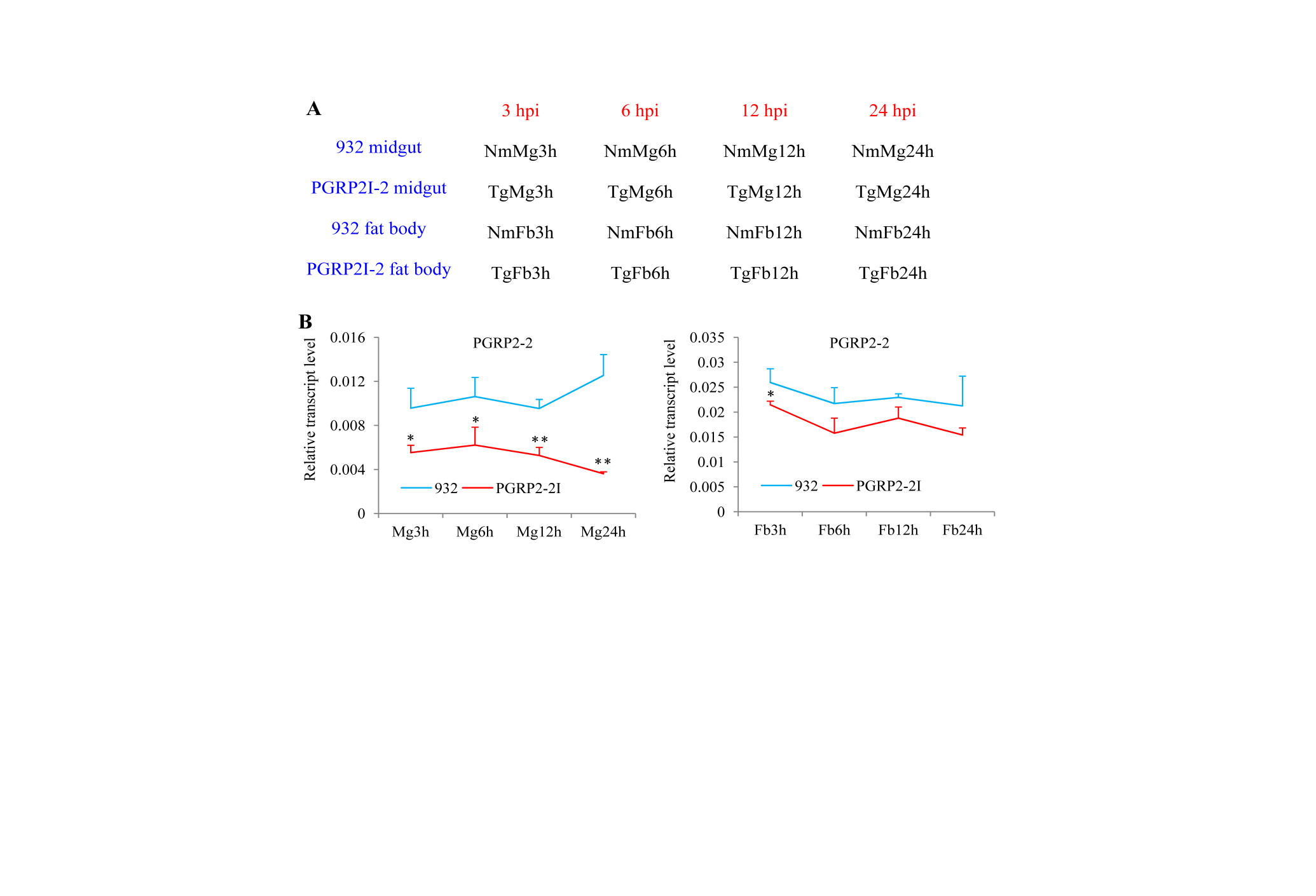


**Figure S9.** Transcriptome analysis. (A) Sample preparation. RNA was extracted from midgut and fat body tissues of PGRP2-2I and 932 at 3, 6, 12, and 24 hpi. (B) qPCR analysis of *BmPGRP2-2* expression. *TIF-4A* was used as a control, and each assay was performed thrice. Student’s *t*-tests were used to analyze the statistical data. Bars represent standard deviation. **P* < 0.05, ***P* < 0.01.

**
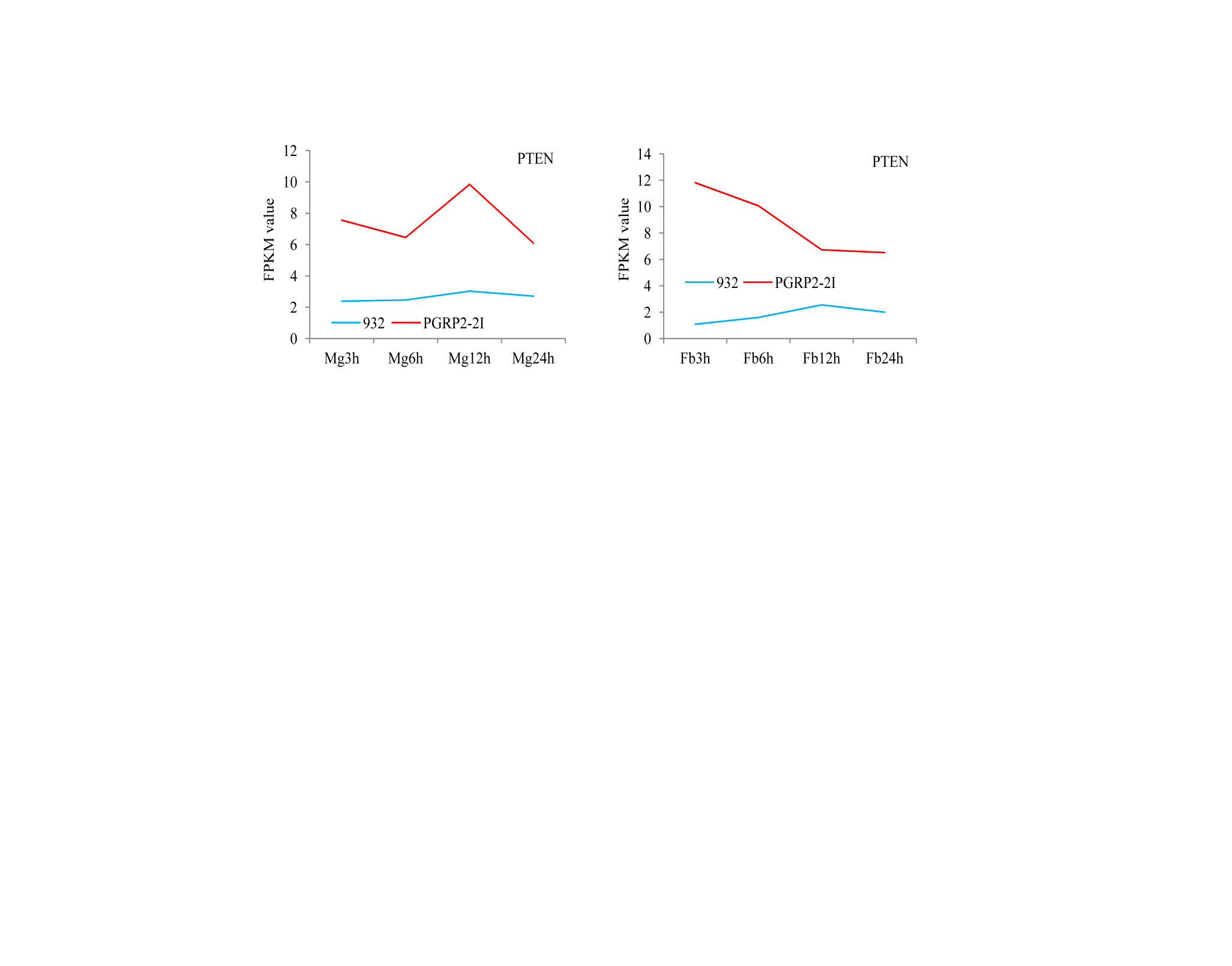
**

**Figure S10.** Fragments per kilobase of exon per million fragments mapped (FPKM) value of *PTEN* from the transcriptome analysis. Values obtained by RNA sequencing are shown in Supplementary Materials 2.


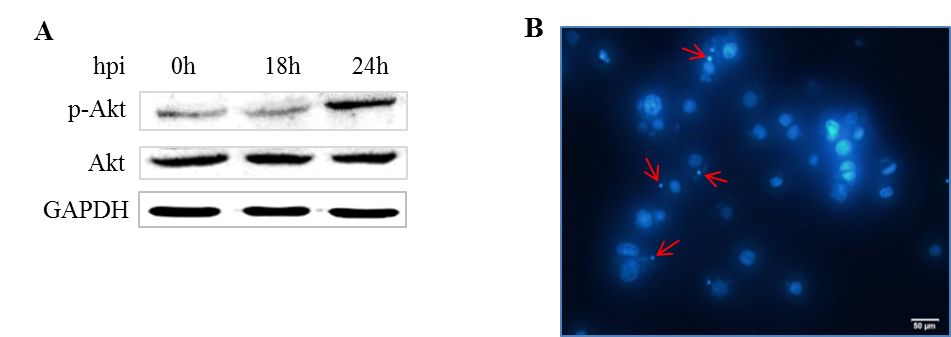


**Figure S11.** BmNPV infection induces (A) Akt phosphorylation and (B) formation of apoptotic bodies.

**Table. S1**. Primers (designing with Primer premier 5.0)

|  | |  | |  | |
| --- | --- | --- | --- | --- | --- |
| Primer name | Forward primer | | Reverse primer | | Genes |
| BmPGRP2-1 ORF | 5' ATGAAGAGTGATGGTGATGAAAAT 3' | | 5' TTAACCCCGGAGACAAAGA 3' | | BmPGRP2-1 |
| BmPGRP2-2 ORF | 5' ATGAAGAGTGATGGTGATGAAAAT 3' | | 5' TTAGTTTTCACTTTCGATCATTG 3' | | BmPGRP2-2 |
| 5F-1 | 5' ATAAATCTATGCGTTCTACGA 3' | |  | | 5ꞌ UTR |
| 5F-2 | 5' GAATTACGTGATAGCTCATTT 3' | |  | | 5ꞌ UTR |
| 5F-3 | 5' AAAACAACATTTAATGCCC 3' | |  | | 5ꞌ UTR |
| 5F-4 | 5' TTCAACTGGTTCTTGGAA 3' | |  | | 5ꞌ UTR |
| 5F-5 | 5' TGCATTACAATCACAGTCG 3' | |  | | 5ꞌ UTR |
| 5F-6 | 5' AATTGGGCATTATGGTGT 3' | |  | | 5ꞌ UTR |
| 5R-1 |  | | 5' AGCCCAGTCCTAATGTGA 3' | | 5ꞌ UTR |
| BmPGRP2-1qRT | 5' TCGCCATACTCAGTGGATTTC 3' | | 5' TACGATGCCGTTACCAGACA 3' | | BmPGRP2-1 |
| BmPGRP2-2qRT | 5' TAATTGGCAACGACAATAGAG 3' | | 5' AGTAATCGGGATGTAGGAAAC 3' | | BmPGRP2-2 |
| Bmglv2 | 5' ATTACTCGATCAGCGGGCAATCC 3' | | 5' GCCGTAGTTGGTGCTGTCG 3' | | Bmglv2 |
| Bmatt2 | 5' AGATGTCCAAGAGTGTAGCGT 3' | | 5' CCCATTATCAAGATTATTTAGAAG 3' | | Bmatt2 |
| Bmimd | 5' CGAAGAAGTTATCATTGAGGAA 3' | | 5' TTATGGTTGTTAGGGTCAGGTT 3' | | Bmimd |
| BmPGRP2-1pro | 5' CGGGATCCTCCTTCGTCATAGTTG-  GTCAC 3' | | 5' ATAAGAATGCGGCCGCTTAACCCC-  GGAGACAAAGAC 3' | | BmPGRP2-1 |
| BmPTEN ORF | 5' ATGGCAAACTCCATGTCAAATA 3' | | 5' TCACGTGTGGTCGGGGG 3' | | BmPTEN |
| BmPTENqRT | 5' CTGATAGTGGAGAAGGTGCCG 3' | | 5' GTAATGGCCGACGCGCT 3' | | BmPTEN |
| GAPDH | 5' CATTCCGCGTCCCTGTTGCTAAT 3' | | 5' GCTGCCTCCTTGACCTTTTGC 3' | | GAPDH |
| TIF-4A | 5' GAATGGACCCTGGGACACTT 3' | | 5' CTGACTGGGCTTGAGCGATA 3' | | TIF-4A |

**Table. S2**. FPKM values of silkworm immune-related genes.

| Gene ID | NmMg3h | NmMg6h | NmMg12h | NmMg24h | TgMg3h | TgMg6h | TgMg12h | TgMg24h | NmFb3h | NmFb6h | NmFb12h | NmFb24h | TgFb3h | TgFb6h | TgFb12h | TgFb24h | Gene | Pathway |
| --- | --- | --- | --- | --- | --- | --- | --- | --- | --- | --- | --- | --- | --- | --- | --- | --- | --- | --- |
| BGIBMGA002397 | 2.2 | 3.7 | 3.4 | 2.8 | 3.5 | 3.9 | 4.6 | 5.5 | 22.3 | 9.2 | 7.1 | 7.1 | 9.5 | 10.3 | 10.6 | 9.0 | BmSpz1 | Toll |
| BGIBMGA012697 | 0.0 | 0.0 | 0.1 | 0.1 | 0.0 | 0.0 | 0.1 | 0.0 | 0.2 | 0.0 | 0.2 | 0.1 | 0.9 | 0.2 | 0.8 | 0.1 | BmSpz2 | Toll |
| BGIBMGA010871 | 0.2 | 0.1 | 0.1 | 0.1 | 0.2 | 0.3 | 0.3 | 0.2 | 0.4 | 0.1 | 0.0 | 0.3 | 0.0 | 0.1 | 0.1 | 0.3 | BmSpz3 | Toll |
| BGIBMGA002869 | 12.7 | 13.2 | 14.7 | 14.5 | 13.6 | 13.1 | 15.6 | 15.4 | 3.7 | 10.3 | 13.0 | 12.0 | 12.1 | 13.6 | 11.1 | 15.3 | BmMyD88 | Toll |
| BGIBMGA002869 | 12.7 | 13.2 | 14.7 | 14.5 | 13.6 | 13.1 | 15.6 | 15.4 | 3.7 | 10.3 | 13.0 | 12.0 | 12.1 | 13.6 | 11.1 | 15.3 | BmTube | Toll |
| BGIBMGA000063 | 27.7 | 24.1 | 21.8 | 27.5 | 28.7 | 26.2 | 20.2 | 26.5 | 21.0 | 17.0 | 14.9 | 15.0 | 20.5 | 22.9 | 17.7 | 15.7 | BmPelle | Toll |
| BGIBMGA010496 | 4.7 | 5.0 | 5.3 | 5.1 | 4.2 | 5.0 | 5.6 | 6.3 | 3.7 | 10.3 | 10.0 | 7.9 | 12.0 | 10.7 | 9.6 | 7.7 | BmRelA | Toll |
| BGIBMGA010497 | 2.2 | 2.1 | 2.6 | 2.0 | 1.9 | 2.8 | 2.7 | 3.0 | 2.8 | 7.4 | 6.8 | 5.2 | 9.7 | 7.0 | 6.6 | 5.9 | BmRelB | Toll |
| BGIBMGA006726 | 6.5 | 5.7 | 5.6 | 4.8 | 8.4 | 7.0 | 5.8 | 5.5 | 6.1 | 4.7 | 7.9 | 4.6 | 7.9 | 6.5 | 7.8 | 5.2 | BmDredd | Imd |
| BGIBMGA008980 | 6.2 | 8.2 | 7.8 | 7.5 | 7.1 | 8.2 | 8.1 | 8.5 | 2.5 | 6.3 | 8.2 | 6.3 | 8.0 | 7.6 | 6.9 | 7.8 | BmTAK1 | Imd |
| BGIBMGA008389 | 4.1 | 5.4 | 4.0 | 3.9 | 4.0 | 5.1 | 3.7 | 3.6 | 1.7 | 3.0 | 3.4 | 3.1 | 3.0 | 3.3 | 2.9 | 1.9 | BmIKKβ | Imd |
| BGIBMGA004180 | 56.7 | 40.7 | 44.6 | 38.8 | 68.7 | 49.3 | 43.7 | 38.6 | 4.0 | 5.3 | 4.2 | 5.7 | 5.6 | 5.9 | 3.6 | 5.4 | BmIKKγ | Imd |
| BGIBMGA002464 | 94.8 | 80.0 | 69.6 | 70.6 | 146.6 | 109.1 | 69.1 | 79.3 | 4.3 | 8.5 | 10.6 | 10.4 | 18.0 | 15.8 | 11.6 | 9.5 | BmRelish | Imd |
| BGIBMGA007358 | 12.4 | 13.5 | 16.6 | 21.1 | 11.8 | 13.8 | 12.5 | 19.2 | 4.3 | 15.5 | 12.9 | 12.8 | 12.7 | 12.0 | 9.7 | 13.1 | BmJNK | JNK |
| BGIBMGA004164 | 66.8 | 72.5 | 57.4 | 74.0 | 73.9 | 72.1 | 72.3 | 93.6 | 28.0 | 54.3 | 53.7 | 38.8 | 71.9 | 60.0 | 51.6 | 54.9 | BmJun | JNK |
| BGIBMGA005140 | 70.0 | 67.3 | 62.0 | 80.2 | 75.2 | 65.4 | 62.4 | 74.9 | 7.2 | 12.4 | 13.0 | 12.0 | 16.5 | 19.0 | 14.5 | 17.0 | BmFos | JNK |
| BGIBMGA003997 | 13.2 | 13.2 | 13.9 | 12.2 | 13.1 | 11.9 | 13.2 | 11.8 | 9.8 | 11.1 | 19.2 | 12.5 | 13.0 | 11.4 | 16.6 | 12.0 | BmPIAS | JAK/STAT |
| BGIBMGA009619 | 15.3 | 22.8 | 15.9 | 19.5 | 16.8 | 23.8 | 16.3 | 27.4 | 11.3 | 14.9 | 8.3 | 14.9 | 10.7 | 17.6 | 9.6 | 17.5 | BmSOCS | JAK/STAT |
| BGIBMGA001739 | 16.7 | 13.0 | 12.3 | 10.8 | 15.8 | 14.3 | 12.5 | 11.6 | 2.4 | 9.0 | 9.4 | 7.6 | 14.4 | 11.9 | 9.1 | 8.6 | BmSTAT | JAK/STAT |
| BGIBMGA010561 | 6.0 | 4.4 | 4.8 | 3.7 | 6.3 | 4.4 | 5.5 | 4.1 | 1.2 | 2.6 | 5.2 | 3.2 | 4.0 | 2.7 | 5.1 | 3.0 | BmPI3K | PI3K/Akt |
| BGIBMGA008304 | 12.7 | 12.2 | 10.8 | 11.2 | 11.8 | 13.1 | 12.6 | 13.1 | 1.7 | 16.4 | 15.4 | 12.0 | 15.2 | 22.7 | 11.0 | 13.8 | BmAkt | PI3K/Akt |
| BGIBMGA009071 | 2.4 | 2.5 | 3.0 | 2.7 | 7.6 | 6.5 | 9.8 | 6.1 | 1.1 | 1.6 | 2.6 | 2.0 | 11.8 | 10.1 | 6.7 | 6.5 | BmPTEN | PI3K/Akt |
| BGIBMGA012763 | 0.2 | 0.2 | 0.5 | 0.5 | 0.4 | 0.3 | 0.6 | 0.5 | 1.1 | 5.9 | 14.5 | 7.0 | 4.7 | 3.6 | 11.0 | 6.7 | BmPPO1 | PPO |
| BGIBMGA013115 | 0.9 | 1.6 | 2.7 | 1.9 | 1.4 | 1.4 | 2.4 | 2.7 | 6.5 | 4.1 | 11.9 | 5.6 | 5.9 | 5.8 | 9.4 | 6.8 | BmPPO2 | PPO |
